# Delineating the drought vulnerability zones in Bangladesh

**DOI:** 10.1038/s41598-024-75690-w

**Published:** 2024-10-26

**Authors:** Showmitra Kumar Sarkar, Swadhin Das, Rhyme Rubayet Rudra, Khondaker Mohammed Mohiuddin Ekram, Mafrid Haydar, Edris Alam, Md Kamrul Islam, Abu Reza Md. Towfiqul Islam

**Affiliations:** 1 Department of Urban and Regional Planning, Khulna University of Engineering & Technology, Khulna, 9203 Bangladesh; 2 Population Health Sciences, Harvard University, Harvard, USA; 3 Faculty of Resilience, Rabdan Academy, Abu Dhabi, 22401 United Arab Emirates; 4 Department of Geography and Environmental Studies, University of Chittagong, Chittagong, 4331 Bangladesh; 5 Department of Civil and Environmental Engineering, College of Engineering, King Faisal University, AlAhsa, 31982 Saudi Arabia; 6 Department of Disaster Management, Begum Rokeya University, Rangpur, 5400 Bangladesh; 7 Department of Development Studies, Daffodil International University, Dhaka, 1216 Bangladesh

**Keywords:** Drought, Agriculture, Analytic hierarchy process, GIS, Bangladesh, Climate sciences, Environmental sciences, Natural hazards

## Abstract

The research aims to explore the vulnerability of Bangladesh to drought by considering a comprehensive set of twenty-four factors, classified into four major categories: meteorological, hydrological, agricultural, and socioeconomic vulnerability. To achieve this, the study utilized a knowledge-based multi-criteria method known as the Analytic Hierarchy Process (AHP) to delineate drought vulnerability zones across the country. Weight estimation was accomplished by creating pairwise comparison matrices for factors and different types of droughts, drawing on relevant literature, field experience, and expert opinions. Additionally, online-based interviews and group discussions were conducted with 30 national and foreign professionals, researchers, and academics specializing in drought-related issues in Bangladesh. Results from overall drought vulnerability map shows that the eastern hills region displays a notably high vulnerability rate of 56.85% and an extreme low vulnerability rate of 0.03%. The north central region shows substantial vulnerability at high levels (35.85%), while the north east exhibits a significant proportion (41.68%) classified as low vulnerability. The north west region stands out with a vulnerability rate of 40.39%, emphasizing its importance for drought management strategies. The River and Estuary region displays a modest vulnerability percentage (38.44%), suggesting a balanced susceptibility distribution. The south central and south east regions show significant vulnerabilities (18.99% and 39.60%, respectively), while the south west region exhibits notable vulnerability of 41.06%. The resulting model achieved an acceptable level of performance, as indicated by an area under the curve value of 0.819. Policymakers and administrators equipped with a comprehensive vulnerability map can utilize it to develop and implement effective drought mitigation strategies, thereby minimizing the losses associated with drought.

## Introduction

A situation of dry weather that lasts for a lengthy period and leads to an imbalanced hydrological activity, also known as a lack of available water, is what the term “drought” refers to, according to its definition^1^. Drought is one of the most devastating natural disasters in the world, occurring with different frequencies and severity across a vast array of climatic regions^2–4^. Given that it is characterized by a prolonged period of below-normal rainfall, it is the most difficult of the recurrent severe weather events^5^. A decrease in the amount of precipitation that falls each year and an increase in the amount of evapotranspiration that occurs as a direct result of a rise in temperature, or any combination of the two, are the primary factors that contribute to water scarcity^1^.

Drought has a significant impact on the environment, society, economy, agriculture, and natural and water resources^6^. Drought-related catastrophes produce more economic losses and have a greater negative effect on human civilization than any other climatic disaster^7^. Droughts are expected to become more common and severe as a result of climate change and human activity, according to recent research^8–10^. Several regions of the earth often face recurring drought situations, which harm a lot of people every year^11^. Over 410 major droughts occurred around the globe between 1980 and 2008, impacting 53.5 million people annually^4^. The drought-related calamity results in annual economic losses of between US$6 and US$8 billion worldwide^3,10,12^. The increasing frequency and severity of droughts have an impact on many tropical areas in Southeast Asia, Africa, the northeastern region of Brazil, and Australia^4^.

Due to their complexity and the multiplicity of factors that affect them, droughts are challenging to monitor^13^. In general, drought may be broken down into four categories: meteorological drought, agricultural drought, hydrological drought, and socioeconomic drought^14^. Generally, a meteorological drought is a time with abnormally low precipitation levels compared to a region’s long-term average circumstances^15^. A region experiences a meteorological drought when dry weather patterns predominate there^14^. Conventionally, meteorological drought is characterized by the level of dryness and duration of the dry period. Geographical variations in atmospheric conditions resulting in insufficient rainfall necessitate the consideration of location-specific definitions of meteorological drought. Agricultural drought addresses the impact of meteorological (or hydrological) drought on agriculture, focusing on deficiencies in precipitation, differences between current and projected evapotranspiration, shortages of soil water, reduced levels of groundwater or reservoirs, and other related factors. Hydrological droughts refer to the effects of inadequate precipitation, especially snowfall, on the availability of surface or groundwater. Socioeconomic definitions of drought include characteristics of meteorological, hydrological, and agricultural drought into the dynamics of supply and demand for certain economic commodities. The distinction of this kind of drought is in its reliance on the dynamics of supply and demand in both time and location to establish its definition or classification^16^.

Bangladesh is one of the most vulnerable nations to natural disasters because of its geographic location and socioeconomic standing^17–23^. This includes but is not limited to floods, cyclones, storm surges, droughts, heatwaves, rising sea levels, and saline intrusion^4,24–35^. While floods and tropical cyclones have the potential to cause widespread devastation, drought has received relatively little attention. Bangladesh experiences droughts on a 2.5-year cycle on average^36^. Bangladesh has experienced severe droughts in 1973, 1978–1979, 1981–1982, 1989, 1992, 1994–1995, 2000, 2006, 2009, 2012, and 2016, respectively, since gaining independence in 1971^37^. Nearly 47 percent of the country’s landmass has been impacted by severe drought occurrences, where 53 percent of the population resides. Agriculture is Bangladesh’s most important economic sector. These droughts had a tremendous impact on the natural resources of the nation, most notably on its forests, fisheries, agricultural output, and other businesses that are dependent on these areas. For instance, the 1995 drought caused a 3.5 × 106 metric tons decrease in the amount of rice and wheat produced^4^. Climate has a significant impact on the agricultural industry; additionally, our environment is more lenient toward agricultural production than the climate of certain other nations throughout the globe^14^. The nation has experienced a wide range of different natural disasters in recent decades, including droughts of various intensities, which are affecting its socio-environmental aspects. Climate change is projected to lead to an increase in droughts^38^, and South Asia is expected to have less rain during the dry season and more rain during the monsoon season^39,40^. Bangladesh is prone to drought because of the unusually low precipitation that it receives from November to May^40,41^. Droughts may strike at any time of the year, although they are most prevalent in the pre-monsoon and post-monsoon seasons^14^.

Based on the nature of water scarcity, mean periods, degree of truncation, and regionalization techniques, drought can be assessed^4^. Implementing drought management methods requires spatial data on the variables influencing drought sensitivity, sensitive locations, and the degree of vulnerability^2^. All of these pieces of geographical information may be gleaned via detailed drought vulnerability mapping. The degree and scope of a threat’s influence on a region’s people and environment are measured in terms of vulnerability^42^. An effective drought vulnerability mapping technique considers the selection and mapping of each criterion’s effects on various forms of drought vulnerability.

Geographical Information System (GIS) and remote sensing data have been widely employed for the analysis of drought vulnerability. The efficacy of GIS-based Multiple-Criteria Decision Analysis (MCDA) in identifying drought vulnerability stems from its capacity to combine geographical data with other parameters such as climate, soil, and land use. This integration enables a thorough and visually detailed evaluation of vulnerability^40,43,44^. The substantial geographical coverage, digital accessibility, and multi-temporal and multi-spectral analysis of this data package unquestionably provide its advantages in terms of faster and more precise categorizations^45–54^. Integration of remote sensing with spatial analysis provides an ideal setting for efficiently and expeditiously computing a large amount of data. Remotely sensed imagery serves as vital because it provides up-to-date, detailed information on physical conditions of the land surface, the health of vegetation, and the availability of water. This allows for real-time monitoring and more precise evaluation of drought conditions. Spatial analysis tools allow for the integration and analysis of information from many sources^55^. GIS is mainly needed to integrate and evaluate the combined impacts of the topographical, climatic, and land features of the region to qualitatively assess drought^10,56^. GIS may be used to detect locations that are prone to drought by combining it with remote sensing and auxiliary data^18,45^. On the other hand, multiple-criteria decision analysis (MCDA) provides a range of methods and strategies for assessing and organizing choice issues, weighing the pros and cons of various options, and ranking them in order of importance^13^. To provide precise vulnerability data for developing drought mitigation measures, a multi-criterion-based integrated geographic mapping technique for drought vulnerability should be created. This approach would aggregate all drought categories using an appropriate weighting system^7^.

A set of studies have been reported for mapping drought vulnerability using spatial analysis and remote sensing tools, including the fuzzy comprehensive evaluation framework^57^, the composite drought vulnerability indicator^58^, and others remote sensing incises (i.e., normalized difference vegetation index^59^, normalized difference water index^60^, normalized difference drought index^61^, normalized multi‐band drought index^62^, normalized difference moisture index^4^, evapotranspiration^63,64^. Droughts are complicated occurrences, and the relationships between these four types of droughts are intricate^65^. Most research has either isolated one aspect of drought vulnerability mapping^6,66^ or combined two or three aspects using narrow criteria^9,67^. Despite the limited number of research that have encompassed all four categories of drought vulnerability, the focus of these studies has largely been confined to local or regional scales^7,68,69^. To the best of our understanding, studies have successfully integrated all four categories of data on a national level. The objective of this study is to systematically map the vulnerability to drought in Bangladesh, including four different types of droughts. Given the absence of extensive research on drought sensitivity zones in Bangladesh on a national level, this work is of great significance. With the difficulty of using single source drought indices to a multi-dimensional problem, this work states an integrated approach that combines remote sensing-based indices, climate and meteorological data, geological settings, and field observations to quantify the current drought status and assess the potential vulnerability of drought. This study’s key novelty is the manner in which it integrates a wide range of 24 multi-dimensional factors using the Analytic Hierarchy Process (AHP) to produce a thorough, spatially explicit map of Bangladesh’s drought vulnerability that is based on expert insights and customized for the country’s specific circumstances. The research has the following specific objectives: (a) to prepare drought vulnerability factors and sub-factors for different drought types (i.e., metrological, hydrological, agricultural, and socioeconomic), (b) to assess the influences of different factors and sub-factors and measure different types of drought vulnerability, and (c) to develop the drought vulnerability of Bangladesh by integrating different drought types and validate results. The research results can assist farmers, governments, and local authorities in identifying areas at high risk of drought, highlighting focused interventions, optimizing water use, enhancing crop planning, and creating drought mitigation plans tailored to the region to minimize vulnerability and associated losses. This drought vulnerability assessment framework enables other nations to implement comparable catastrophe risk reduction, sustainable resource management, and policy formulation approaches that are consistent with global climate adaption strategies and in line with international climate resilience objectives. Furthermore, this study clearly aligns with Sustainable Development Goals (SDGs) such as SDG 2: Zero Hunger, SDG 6: Clean Water and Sanitation, SDG 11: Sustainable Cities and Communities, SDG 13: Climate Action, and SDG 15: Life on Land.

## Methods and materials

### Geo-environmental setting of the study area

**Fig. 1:**
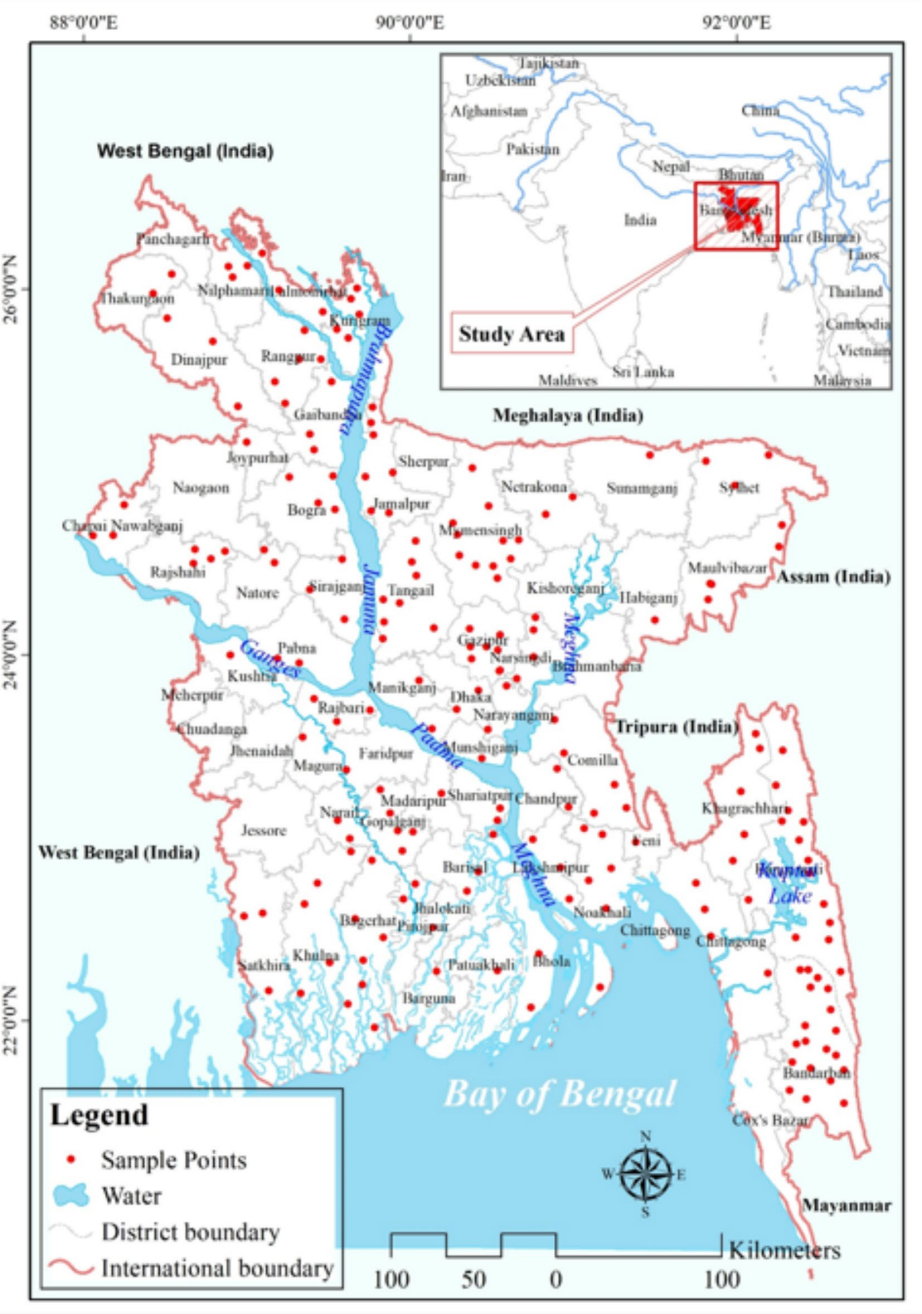
Study area; prepared by the authors using ArcGIS software version 10.5, (https://www.esri.com/en-us/arcgis/products).

This study focuses on Bangladesh (Fig. 1), a country located in South Asia, to analyze the dynamics of drought events and their associated impacts. Drought is a recurring natural hazard in Bangladesh, affecting various sectors such as agriculture, water resources, and socio-economic stability. Bangladesh is situated in the delta of the Ganges–Brahmaputra-Meghna (GBM) river system, bordered by India to the west, north, and east, and Myanmar to the southeast. The country’s topography consists of vast plains, low-lying coastal areas, and riverine networks, making it vulnerable to both meteorological and hydrological droughts. The study area encompasses the entire territory of Bangladesh, including its major cities, rural regions, and coastal areas. Bangladesh experiences a subtropical monsoon climate, characterized by high temperatures, heavy rainfall during the monsoon season (June to September), and relatively dry winters (November to February). The geological composition of Bangladesh is predominantly alluvial, with 80% of its territory consisting of deltaic sediments derived from the Ganges–Brahmaputra-Meghna River system. The topography is predominantly characterized by low height, with 80% of the land being below 10 m and the highest point reaching 1,052 m^70–72^. The region exhibits a tropical monsoon climate characterized by an annual precipitation of 2,200 mm and temperatures ranging from 10 °C to 35 °C. Principal crops include rice (which accounts for 75% of the cultivated land), jute, wheat, maize, and legumes^73,74^. However, the region is prone to drought due to inadequate precipitation, erratic rainfall patterns, prolonged dry spells, and changing climatic patterns events. Droughts in Bangladesh have severe consequences for agriculture, which is the backbone of the country’s economy. The study assesses the impacts of drought on crop production, livestock, and rural livelihoods. It examines the vulnerability of different crops to drought stress, the strategies adopted by farmers to cope with droughts, and the overall implications for food security and rural development. Droughts significantly affect the availability of water resources, especially in densely populated urban areas. Droughts have broader socio-economic impacts on the population, including reduced income opportunities, increased food prices, migration, and conflicts over scarce resources.

### Description of the data

This research project focused on assessing drought vulnerability by examining 20 key factors associated with various types of droughts. These factors were gathered from a variety of reliable secondary sources, including the United States Geological Survey, Geological Survey of Bangladesh, and Bangladesh Meteorological Department, among others (Table 1). Land surface temperature (LST) affects animals and ecosystems locally and globally. This is because manmade surfaces absorb and retain more heat, vegetation cover decreases, human heat emissions increase, and wind patterns change, affecting drought conditions. Extracting Land Surface Temperature (LST) data from LANDSAT—8 picture collections using Google Earth Engine (GEE) with precise formulae. For Land Use and Land Cover (LULC) data, first acquire satellite imagery and ground truth data, and then rectify any image distortions. Next, extract features and use a random forest classifier to assign land cover classes. Evaluation of accuracy was conducted by analyzing validation data and extracted land use and land cover (LULC) utilising the GEE. In order to quantify vegetation, the Normalized Difference Vegetation Index (NDVI) measures the difference between near-infrared light (which vegetation strongly reflects) and red light (which vegetation absorbs). Google Earth Engine (GEE) was used to extract NDVI data from LANDSAT-8 images by NDVI = (NIR-RED)/(NIR + RED) formula. Also. Elevation is extracted from NASA Earth Data and Slope, Drainage Density, Flow Accumulation is compiled from Elevation in GEE. Meteorological parameters have been extracted from sources and synthesized in ArcGIS 10.5 utilizing interpolation and reclassification techniques. Additionally, a field-based survey was conducted to facilitate the collection of 200 sample points, as well as to observe real-life scenarios. To estimate the relative importance of these factors, a pairwise comparison matrix was developed. This matrix allowed for the evaluation of factors and sub-factors within different drought types. The construction of the matrix was informed by a comprehensive review of pertinent literature, combined with insights derived from field experience and expert opinions. To enrich the study, online-based interviews and group discussions were organized, involving 30 national and international professionals, researchers, and academics who possess extensive expertise in addressing drought-related issues specific to Bangladesh. This collaborative approach ensured the inclusion of diverse perspectives and fostered a comprehensive understanding of the subject matter. By integrating spatial data such as satellite images and other relevant information, alongside field-based observations and expert insights, this research project aimed to provide a robust framework for assessing drought vulnerability. The meticulous collection and analysis of data from reputable sources, combined with the valuable input of experienced professionals, researchers, and academics, contribute to the credibility and reliability of the findings.

**Table 1:** Comprehensive description and sources of the used datasets for drought vulnerability zones delineation.

Description of layer	Data sources	Type of the data	Spatial resolution
Rainfall	Bangladesh Meteorological Department	-	-
Specific Humidity	Bangladesh Meteorological Department	-	-
Land Surface Temperature	Compiled From Landsat − 8	Raster	30 m
Evapotranspiration	Bangladesh Meteorological Department	-	-
Curvature	Compiled From DEM	Raster	30 m
Land Cover	Compiled From Landsat − 8	Raster	30 m
Soil Type	Geological Survey of Bangladesh	-	-
Soil Texture	Geological Survey of Bangladesh	-	-
Geology	Geological Survey of Bangladesh	-	-
Morphology	Geological Survey of Bangladesh	-	-
Normalized Difference Vegetation Index	Compiled From Landsat − 8	Raster	30 m
Crop Intensity	Bangladesh Agricultural Development Corporation	Vector	-
Most used Irrigation system	Bangladesh Agricultural Development Corporation	Vector	-
Elevation	NASA Earth Data-SRTM	Raster	30 m
Slope	Compiled From DEM	Raster	30 m
Drainage Density	Compiled From DEM	Raster	30 m
Flow Accumulation	Compiled From DEM	Raster	30 m
Groundwater Level	Geological Survey of Bangladesh	Vector	-
Population Density	Bangladesh Bureau of Statistics	-	-
Household With Tube Well	Bangladesh Bureau of Statistics	-	-
Poor People	Bangladesh Bureau of Statistics	-	-
Agriculture Dependency	Bangladesh Bureau of Statistics	-	-
Irrigated Land	Bangladesh Agricultural Development Corporation	Vector	-
Crop Area	Bangladesh Agricultural Development Corporation	Vector	-
Drought Inventory	Field Survey (200 Sample Points)	Vector	-

### Analytical method

A knowledge-based multi-criteria method (i.e., Analysis Hierarchy Process (AHP)) was applied for delineating drought vulnerability zones in Bangladesh. Using mathematical and psychological concepts, GIS-based vulnerability mapping with AHP is a popular approach in the study of multi-criteria decisions^7^. A total of 24 drought vulnerability indicators were utilized for drought vulnerability mapping (6 for socio-economic, 4 for meteorological, 5 for hydrological, and 9 for agricultural). Each factor was then arbitrarily divided into four/ five sub-factors, and factor maps were constructed, so as to conduct preliminary analysis. Weight for sub-factors were given 1 to 5 based on previous literature. Identifying and fixing logical flaws in AHP is facilitated by the hierarchy of factors utilizing pairwise comparisons^75,76^. There were comparisons made between pairs of factors. It was decided to use weights (same significance, more importance, and less important)^77,78^ to differentiate between two factors. The subjects’ weights were rated on a continuous 9-point scale (1/9, 1/8, 1/7, 1/6, 1/5, 1/4, 1/3, 1/2, 1, 2, 3, 4, 5, 6, 7, 8, 9)^79,80^. Both the eigenvalues and consistency ratio (CR) were computed using the provided weight rating. The following Eq. 1 was used to get the CR^81,82^.

1 $${\text{CR}} = \frac{{{\text{CI}}}}{{{\text{RI}}}}$$

where, RI = mean consistency index; CI = consistency index.

The consistency index (CI) was calculated using the following Eq. 2.

2 $${\text{CI}} = \frac{{\uplambda _{\max } - {\text{N}}}}{{({\text{N}} - 1)}}$$

where, λ_max_ = the largest eigenvalue; N = order of the comparison matrix

3 $${\text{Drought}}\,{\text{Vulnerability}} = \mathop \sum \limits_{{{\text{j}} = 1}}^{{\text{n}}} {\text{Wj}}*{\text{w}}_{{{\text{ij}}}}$$

where, W_j_ = weight of causative factor j; w_ij_ = weighted value of class i of causative factor j.

The CR values < 10% were accepted^83^. Four drought vulnerability (i.e., metrological, hydrological, agricultural and socio-economic) maps were generated through weighted overlays of factors based on factors and sub-factors weight (Eq. 3). Finally, drought vulnerability map was developed by integrating the outputs of four drought vulnerability maps and weight. The result will be categorized into five categories based on drought vulnerability values: (a) very high; (b) high; (c) moderate; (d) low; and (e) very low.

### Validation process

In this study, 200 sample points were obtained to determine whether or not there is a drought in that specific area. These sample points were selected randomly from areas throughout Bangladesh, based on an extensive literature review of drought events and their impacts in Bangladesh, and site visits to determine drought and non-drought points. Essentially, these sample points were taken to validate the method’s accuracy by comparing the AHP-derived priorities with real drought occurrences or impacts. The final receiver operating characteristic (ROC) curve was constructed by comparing the AHP-based results to the field drought scenario. However, creating a ROC curve for an Analytic Hierarchy Process (AHP) application, which is typically a multicriteria decision-making method, requires some adaptation. The ROC curve is a graphical representation of sensitivity (y-axis) and specificity (x-axis) at different cut-off points in test data. It is often depicted as a two-dimensional box with axes that range from 0 to 1. The AUC is a statistic that combines the sensitivity and specificity of a test to determine how reliable the test actually is. A diagnostic test can distinguish between the theoretical drought and the actual value in the field if its area under the curve (AUC) is 1. This indicates that the sensitivity and specificity are both at their highest possible levels, with no false positives or negatives. The likelihood of this happening is quite low. In general, the closer AUC is to one, the better the performance on the exam. The square is subdivided into two equal halves of 0.5 square meters each by drawing a diagonal line from (0, 0) to (1, 1). When the ROC is at this line, there is an equal chance that the test will correctly distinguish between drought areas and non-drought areas. The least AUC value should not be 0, but rather 0.5, because AUC = 0 indicates that the test incorrectly classified all participants with drought as negative and all those without drought as positive. Area = 0 can be transformed into area = 1 and a completely inaccurate test into a completely correct one. A confusion matrix was created based on the AHP predictions and the true class labels. the confusion matrix had four categories: true positive (TP), false positive (FP), true negative (TN), and false negative (FN). Sensitivity (True Positive Rate): Sensitivity = TPTP + FN Sensitivity = TP + FNTP​, Specificity (True Negative Rate): Specificity = TNTN + FP Specificity = TN + FPTN. Systemic verification of the threshold was done to generate a range of sensitivity and specificity values. This process helped us to construct points on the ROC curve. Multiple interacting elements, which can have synergistic or antagonistic effects, frequently influence drought vulnerability. Individual parameter validation may not accurately capture the total impact, hence the integrated model accounts for these interconnections. In practical applications, stakeholders and decision-makers are typically more concerned with the overall vulnerability of a region than with the performance of individual components. By validating the final drought map, we hoped to provide information that is immediately applicable and valuable in decision-making processes. Validating each parameter individually can be resource-intensive and time-consuming. Our approach intended to find a balance between giving a thorough assessment and taking into account practical limits, making the study more practicable in real-world circumstances^84^.

**Fig. 2:**
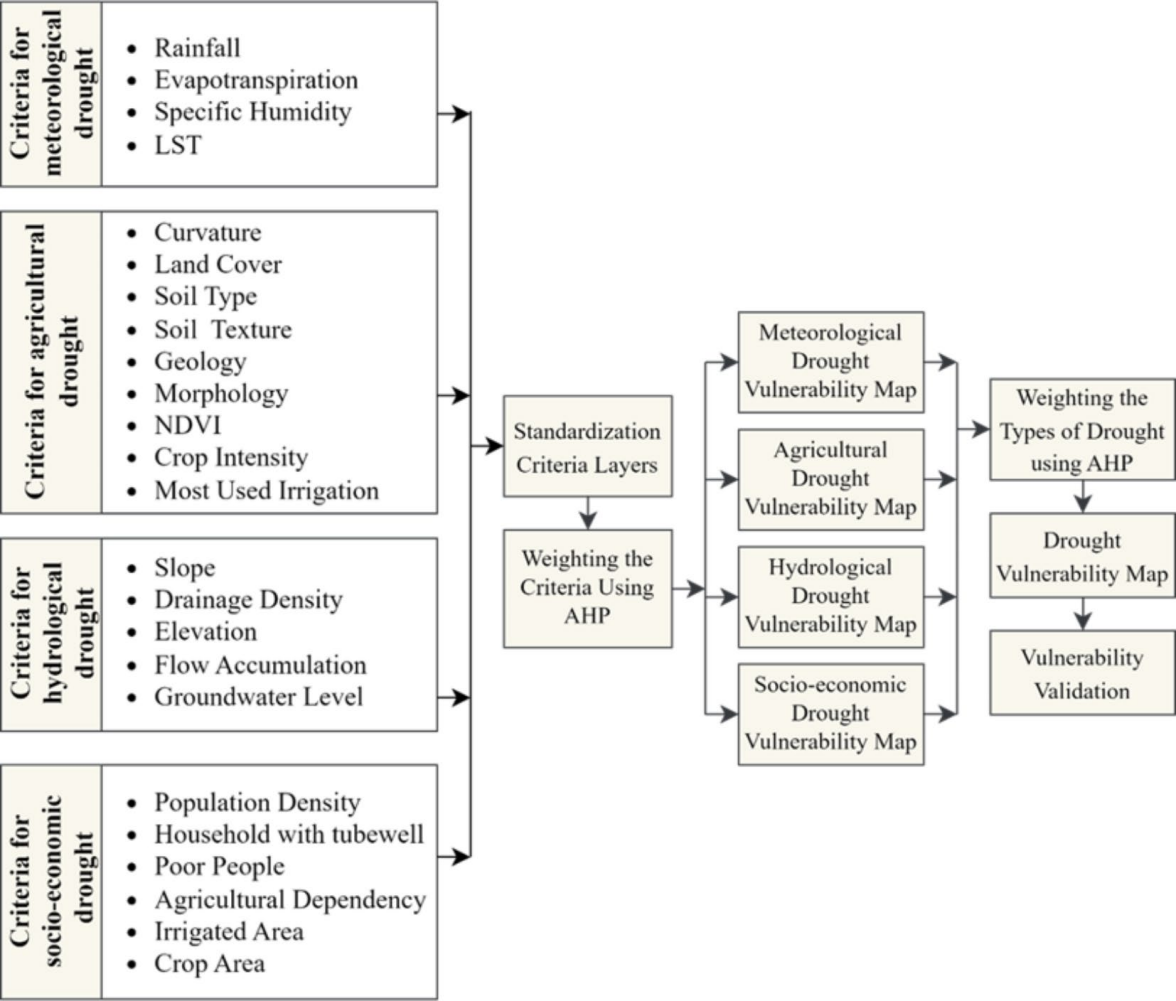
Methodological flow chart of the study.

The methodological flow chart was shown in Fig. 2.

## Results

### Spatial distribution meteorological drought vulnerability factors

**Fig. 3:**
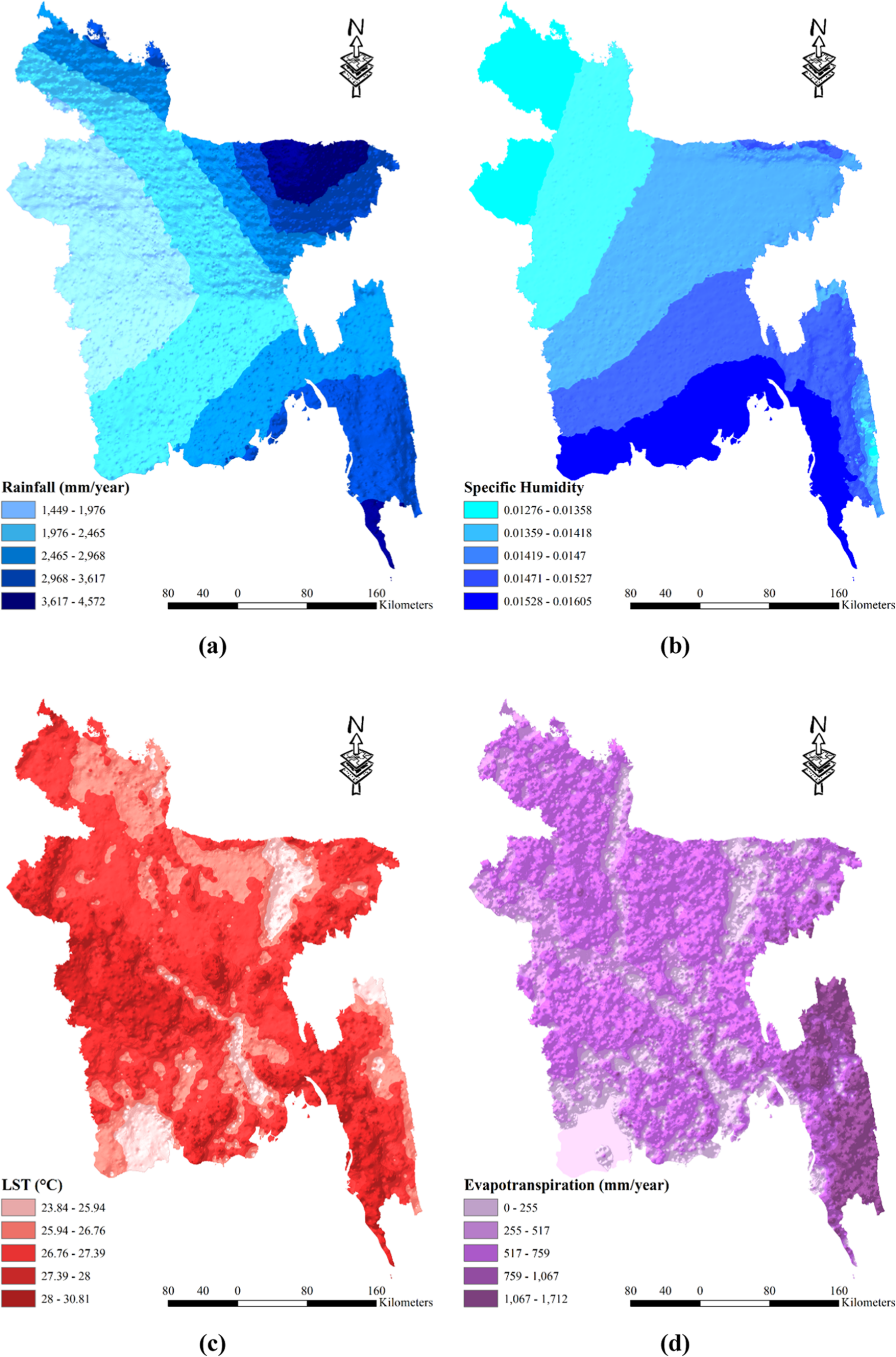
Spatial distribution meteorological drought vulnerability factors: (**a**) Rainfall; (**b**) Specific Humidity; (**c**) Land Surface Temperature; (**d**) Evapotranspiration; prepared by the authors using ArcGIS software version 10.5, (https://www.esri.com/en-us/arcgis/products).

The probability of drought in Bangladesh is inversely related to the amount of rainfall received. Rainfall data from 2001 to 2022 indicates a range of 1449 to 4572 mm per year, which is divided into five zones based on rainfall levels. The zones are as follows: 1449-1976 mm, 1976-2465 mm, 2465-2968 mm, 2968-3617 mm, and 3617-4572 mm. Among the regions, Rajshahi receives the least amount of rainfall, while Sylhet receives the highest (as depicted in Fig. 3 (a)). Higher humidity levels contribute to reduced evaporation and subsequently decrease the likelihood of drought. Specific humidity measurements in Bangladesh from 2001 to 2022 range from 0.01276 to 0.01605 and are categorized into five zones: 0.01276–0.01358, 0.01359–0.01418, 0.01419–0.0147, 0.01471–0.01527, and 0.01528–0.01605. The coastal regions of Bangladesh generally exhibit greater specific humidity compared to the northwestern regions (as illustrated in Fig. 3 (b)). Elevated temperatures exacerbate the occurrence and severity of drought. The average land surface temperature (LST) in Bangladesh during the period from 2000 to 2022 varies from 23.84 °C to 30.81 °C. The LST values are divided into five zones: (23.84–25.94 °C, 25.94–26.76 °C, 26.76–27.39 °C, 27.39–28 °C, and 28–30.81 °C). Regions near Jashore, Chuadanga, and Meherpur experience comparatively higher temperatures (as shown in Fig. 3 (c)). Drought-prone areas often exhibit higher rates of evapotranspiration. The average evapotranspiration (ET) in Bangladesh between 2001 and 2022 ranges from 0 to 1712 mm per year and is classified into five zones: (0–255 mm, 255–517 mm, 517–759 mm, 759–1067 mm, and 1067–1712 mm). Hilly regions, characterized by a greater amount of vegetation, tend to have higher rates of evapotranspiration (as depicted in Fig. 3 (d)). Overall, understanding the interplay between rainfall, humidity, temperature, and evapotranspiration is crucial for assessing the probability of drought in different regions of Bangladesh. These factors provide valuable insights for developing effective drought mitigation strategies and resource management practices in the country.

### Spatial distribution agricultural drought vulnerability factors

**Fig. 4:**
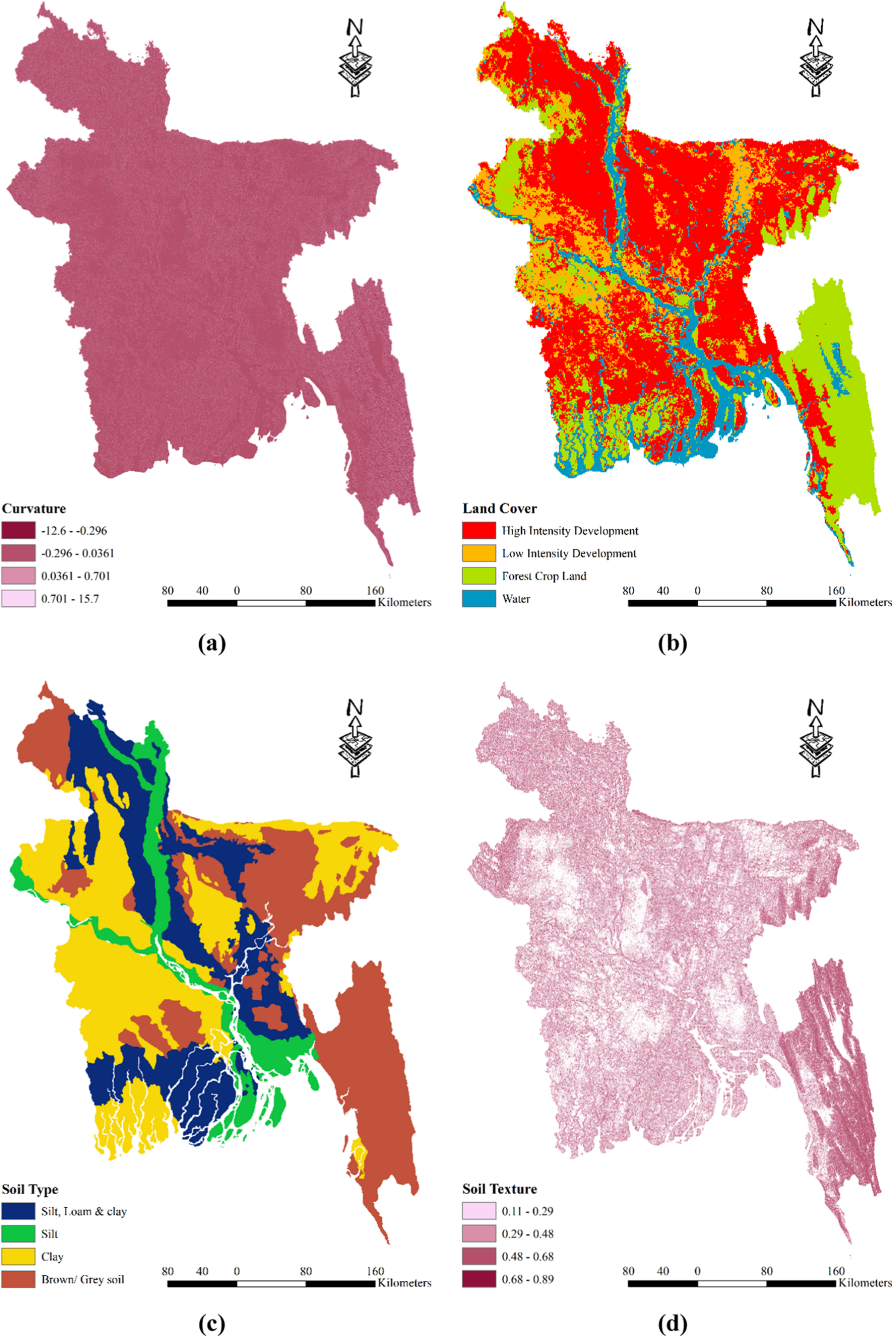

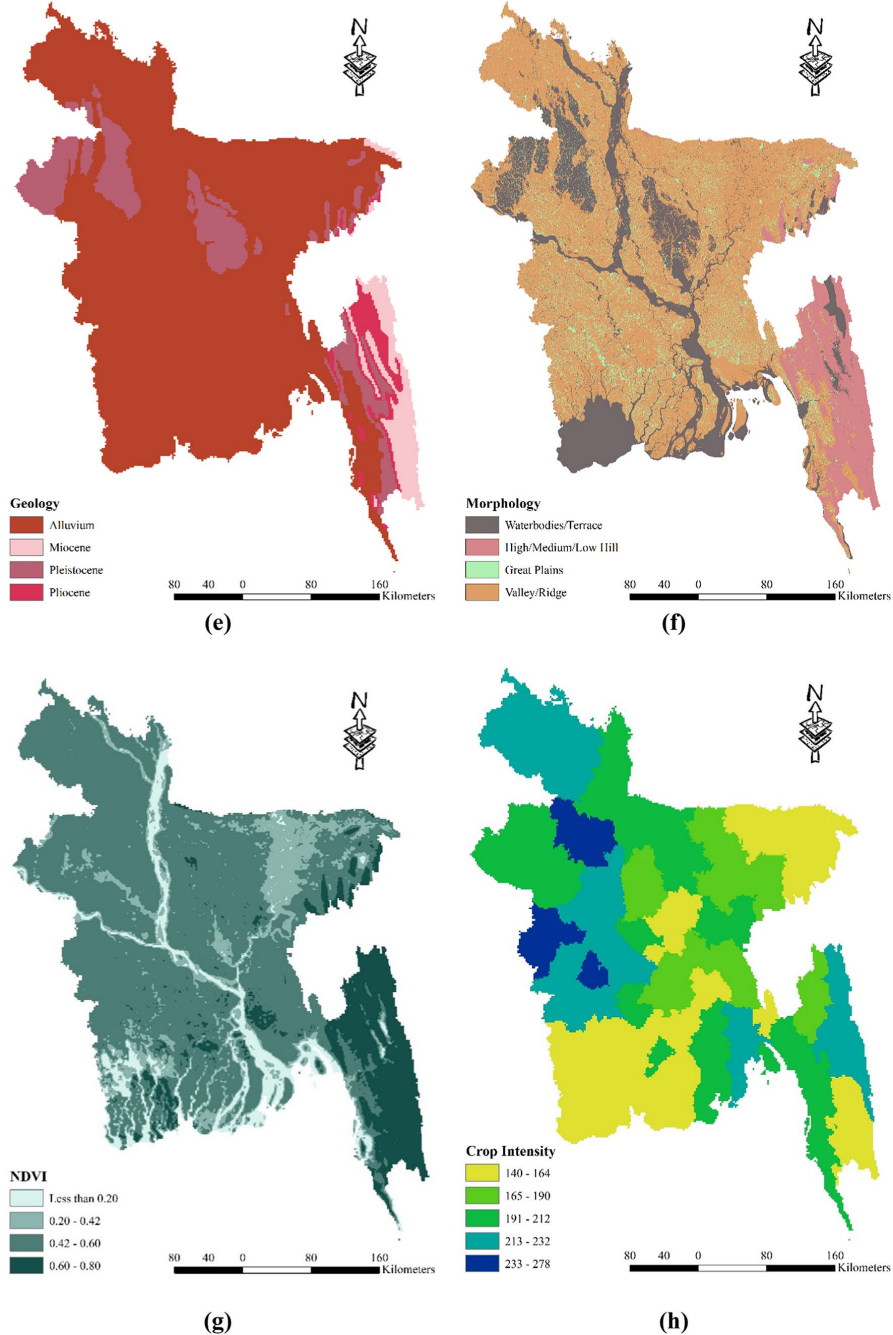

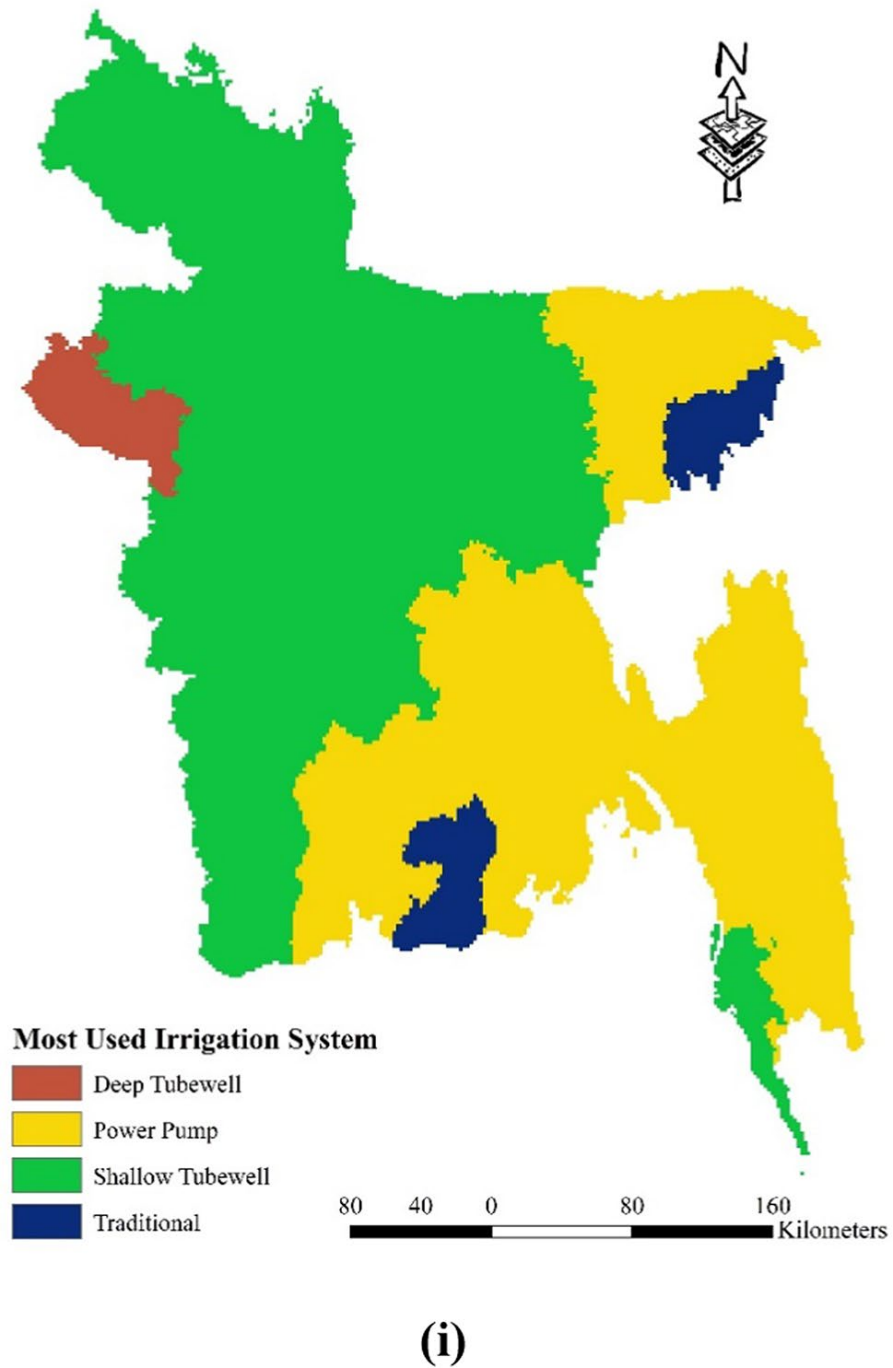
Spatial distribution agricultural drought vulnerability factors: (**a**) Curvature; (**b**) Land Cover; (**c**) Soil Type; (**d**) Soil Texture; (**e**) Geology; (**f**) Morphology; (**g**) Normalized Difference Vegetation Index (**h**) Crop Intensity (**i**) Most used Irrigation system; prepared by the authors using ArcGIS software version 10.5, (https://www.esri.com/en-us/arcgis/products).

Bangladesh exhibits a varying curvature across its landscape, ranging from -12.6 to 15.7. To classify these curvature values, four distinct classes are employed: -12.6 to -0.296, -0.296 to 0.0361, 0.0361 to 0.701, and 0.701 to 15.7 (as depicted in Fig. 4 (a)). When considering land cover categories, high-intensity development is prevalent throughout the country, while forested areas are primarily concentrated in the southeastern part of Bangladesh, encompassing the Chittagong Hill Tracts. In districts like Meherpur, Chuadanga, and Kushtia (as shown in Fig. 4 (b)), one can observe low-intensity development. The land cover also includes water bodies. Examining the soil types within the study area, Bangladesh comprises predominantly silt, loam, clay, and brown/gray soils. However, the proportion of silt and clay is relatively lower. Clay soil can be found in districts such as Kushtia, Rajbari, Faridpur, Magura, Jessore, Meherpur, Chuadanga, and Sylhet. Grey soil is prominent in the Chittagong division and districts like Sunamganj, Habiganj, Thakurgaon, and Panchagarh. Additionally, the districts adjacent to the Jamuna River feature a combination of silt, loam, and clay soils (as illustrated in Fig. 4 (c)). The soil texture in Bangladesh, represented in Fig. 4 (d), exhibits a range from 0.11 to 0.89 and is classified into four zones: 0.11–0.29, 0.29–0.48, 0.48–0.68, and 0.68–0.89 (Table 2). Notably, the hilly regions of Bangladesh demonstrate higher soil texture values. Geographically, Bangladesh comprises various classes, including alluvium, Miocene, Pleistocene, and Pliocene, as shown in Fig. 4 (e). The hilly region predominantly consists of Miocene soil, whereas alluvium types are prevalent throughout the country. Pleistocene soil can be found in districts such as Nawabganj, Rajshahi, Gazipur, and Narsingdi. In terms of morphology, Bangladesh encompasses different classes such as water bodies, hills, vast plains, and valleys. The hills are primarily located in the southeast region, while the majority of the country features valleys and ridges. Water bodies and terraces are prominent in the main rivers and the southwestern part of the region (as depicted in Fig. 4 (f)). Figure 4 (g) also presents the Normalized Difference Vegetation Index (NDVI. Positive NDVI values indicate areas with dense vegetation, while near-zero or negative values represent water and built-up areas. NDVI is an efficient indication of vegetative stress, making it useful for monitoring crops. NDVI values are connected with agricultural drought vulnerability because they show unhealthy vegetation. In contrast, during drought circumstances, water stress impairs plant physiology, resulting in lower water content, chlorophyll activity, and, as a result, lower NDVI values^85^. In the context of drought vulnerability, low NDVI values indicate the impact of water scarcity on crops, revealing reduced yields and compromised agricultural output. However, the interpretation should be nuanced, taking into account unique crop traits, soil conditions, and environmental influences^86^. The NDVI is categorized into four zones: less than 0.20, 0.2 to 0.42, 0.42 to 0.60, and 0.60 to 0.80. Figure 4 (h) shows the Crop Intensity across Bangladesh. It can be said crop intensity is heterogeneous across the country. Districts like Shylet, Khulna, and Chittagong shows low levels of intensity. Finally, Fig. 4 (i) shows the most used Irrigation system across the country Overall, these various characteristics provide valuable insights into the diverse environmental features of Bangladesh, including its curvature, land cover, soil types, soil texture, geographic classes, and morphological classes.

**Table 2:** Alternative ranking scheme based on the contribution to the drought vulnerability.

Components	Criteria	Ranking (based on vulnerability)				
		Very Low (1)	Low (2)	Moderate (3)	High (4)	Very High (5)
Meteorological Drought	Rainfall (mm)	4572–3617	3617–2968	2968–2465	2465–1976	1976–1449
	Evapotranspiration (mm)	0–255	255–517	517–759	759–1067	1067–1712
	Specific Humidity (1 (mass fraction))	0.0160–0.0152	0.0152–0.0146	0.0146–0.0141	0.0141–0.0135	0.0135–0.0127
	LST (°C)	23.83–25.94	25.94–26.76	26.76–27.39	27.39–27.99	27.99–30.81
Agricultural Drought	Curvature	-12.3–0.296	-0.296–0.0361	-	0.0361–0.701	0.701–15.7
	Land Cover	Water	High-Intensity Development	-	Low-Intensity Development	Forest/Crop Land
	Soil Type	Silt	Clay	-	Silt, Loam & Clay	Brown/ Grey Soil
	Soil Texture	0.11–0.29	0.29–0.48	-	0.48–0.68	0.68–0.89
	Geology	Alluvium	Pliocene	-	Miocene	Pleistocene
	Morphology	Waterbodies	Valley/Ridge	-	Great Plains	High/Medium/Low Hill
	NDVI	0.60–0.80	0.42–0.60	-	0.20–0.42	Less than 0.20
	Crop Intensity	140–164	164–195	-	195–220	220–278
	Most Used Irrigation	Traditional	Shallow Tubewell	-	Deep Tubewell	Power Pump
Hydrological Drought	Slope (°)	0–1.62	1.62–5.42	5.42–11.94	11.94–21.44	21.44–69.20
	Drainage Density (Km/Km^2^)	1.01–0.65	0.65–0.49	0.49–0.39	0.39–0.27	0.27–0
	Elevation (m)	0–32	32–109	109–245	245–464	464–1028
	Flow Accumulation	27,590,924–17,744,751	17,744,751–11,685,567	11,685,567–5,518,184	5,518,184–1,190,196	1,190,196–0
	Groundwater Level (m)	0–5.14	5.14–8.51	8.51–14.98	14.98–32.59	32.59–66.52
Socio-economic Drought	Population Density (sq.km)	104–376	376–1033	1033–1807	1807–5560	5560–9972
	Household With Tube Well (%)	98–94	94–89	89–75	75–41	41–31
	Poor People (%)	3–17	17–28	28–35	35–44	44–64
	Agriculture Dependency (%)	75–67	67–59	59–47	47–17	17–4
	Irrigated Land (%)	97.1–59.8	59.8–49.2	49.2–38.3	38.3–25	25–7.36
	Crop Area (Acre)	1,720,000–1,065,000	1,065,000–732,000	732,000–476,000	476,000–249,000	249,000–118,000

### Spatial distribution hydrological drought vulnerability factors

**Fig. 5:**
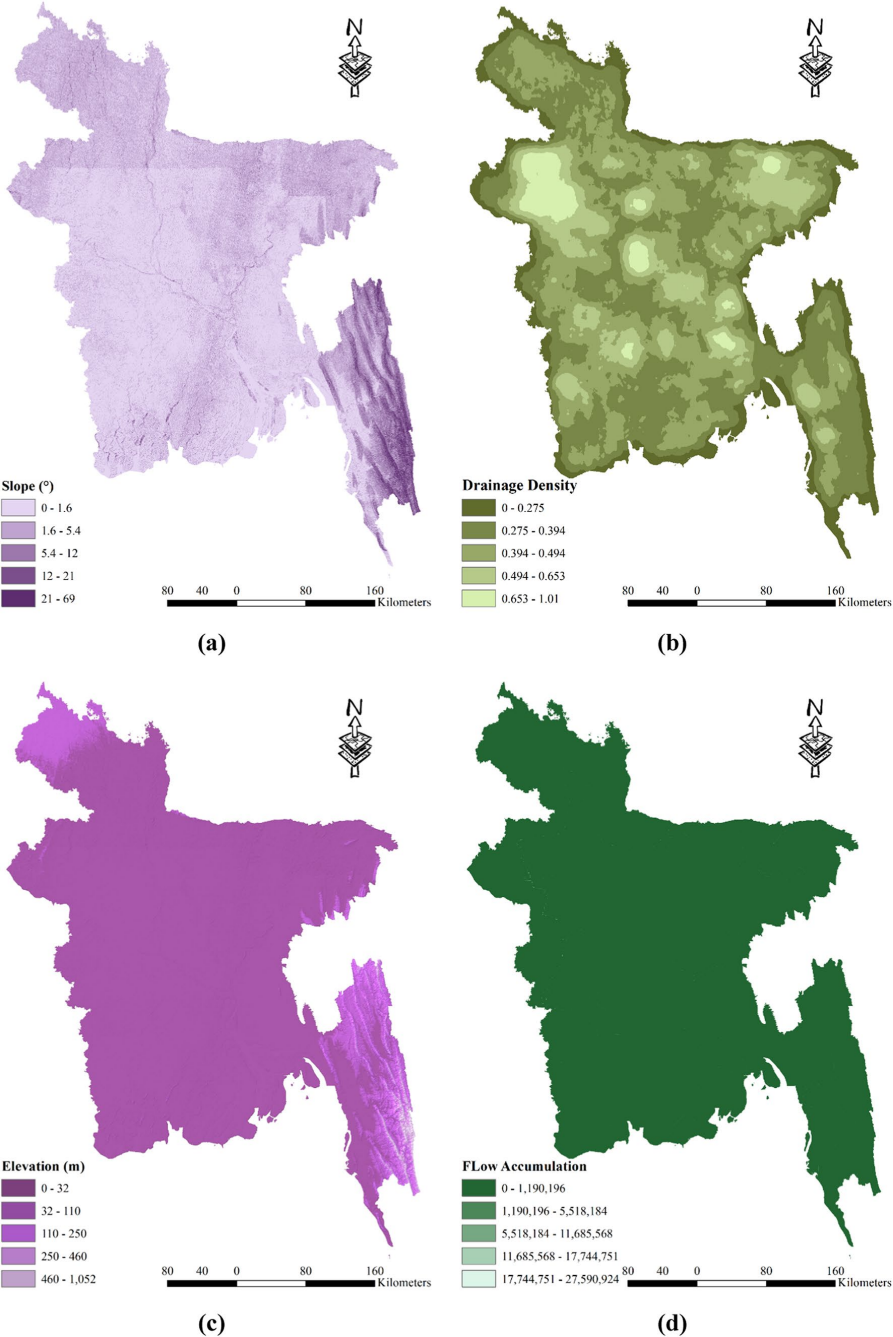

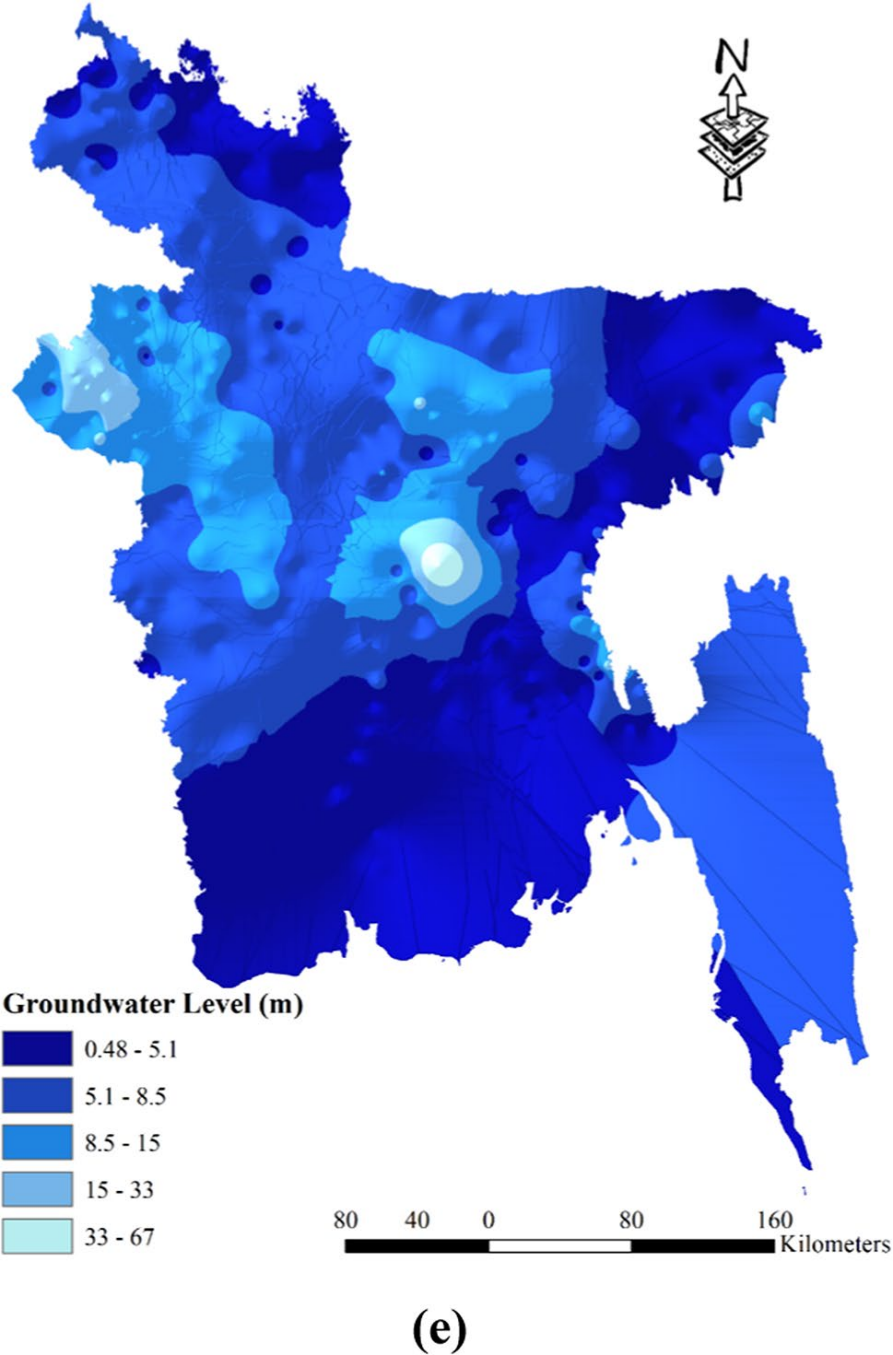
Spatial distribution hydrological drought vulnerability factors: (**a**) Slope; (**b**) Drainage Density; (**c**) Elevation; (**d**) Flow Accumulation; (**e**) Groundwater Level; prepared by the authors using ArcGIS software version 10.5, (https://www.esri.com/en-us/arcgis/products).

The severity of drought in Bangladesh can be influenced by various hydrological factors. One such factor is the slope of the land, which plays a significant role. Steeper slopes tend to exacerbate drought conditions. In Bangladesh, slopes are classified into five zones based on their angle: 0–1.6°, 1.6–5.4°, 5.4–12°, 12–21°, and 21–69°. Notably, the hilly areas of Chittagong exhibit a steeper slope compared to other parts of the country (refer to Fig. 5 (a)). Another contributing factor is the drainage density, as illustrated in Fig. 5 (b). Locations with lower drainage density are more prone to experiencing drought. Drainage density is categorized into five zones: 0–0.275, 0.275–0.394, 0.394–0.494, 0.494–0.653, and 0.653–1.01. The western part of the country, encompassing districts like Naogoan, Rajshahi, and Natore, has relatively high drainage density. Similarly, districts in the eastern part, including Sunamgonj, Sylhet, and Habigonj, also exhibit high drainage density. However, the districts in the Chittagong division, especially those in the hilly tracks, have lower levels of drainage density. Elevation is yet another influential factor in drought susceptibility, with higher-elevation regions being more prone to drought. In Bangladesh, elevation is categorized into five classes: 0–32 m, 32–110 m, 110–250 m, 250–460 m, and 460–1052 m (see Fig. 5 (c)). The Chittagong region, characterized by its higher altitude, is particularly susceptible to drought due to its elevation. Lastly, the likelihood of drought decreases as flow accumulation increases, as depicted in Fig. 5 (d). Flow accumulation is classified into five zones: 0–1,190,196, 1,190,196–5,518,184, 5,518,184–11,685,568, 11,685,568–17,744,751, and 17,744,751–27,590,924 (Table 2). Therefore, areas with higher flow accumulation are less prone to drought incidents. Groundwater levels also play a role in drought susceptibility. In Bangladesh, the groundwater level varies from 0.48 m to 8.5 m and is divided into five zones: 0.48–5.1 m, 5.1–8.5 m, 8.5-15 mm, 15-33 mm, and 33-67 m (Table 2). The vicinity of Dhaka and Rajshahi generally exhibits higher groundwater levels, whereas areas such as Sylhet, the hill tracks of Chittagong, and the coastal region tend to have lower groundwater levels (as illustrated in (Fig. 5 (e)). These various geographical factors, such as slope, drainage density, elevation, groundwater level, and flow accumulation, all contribute to the understanding of drought vulnerability in different regions of Bangladesh.

### Spatial distribution socio-economic drought vulnerability factors

**Fig. 6:**
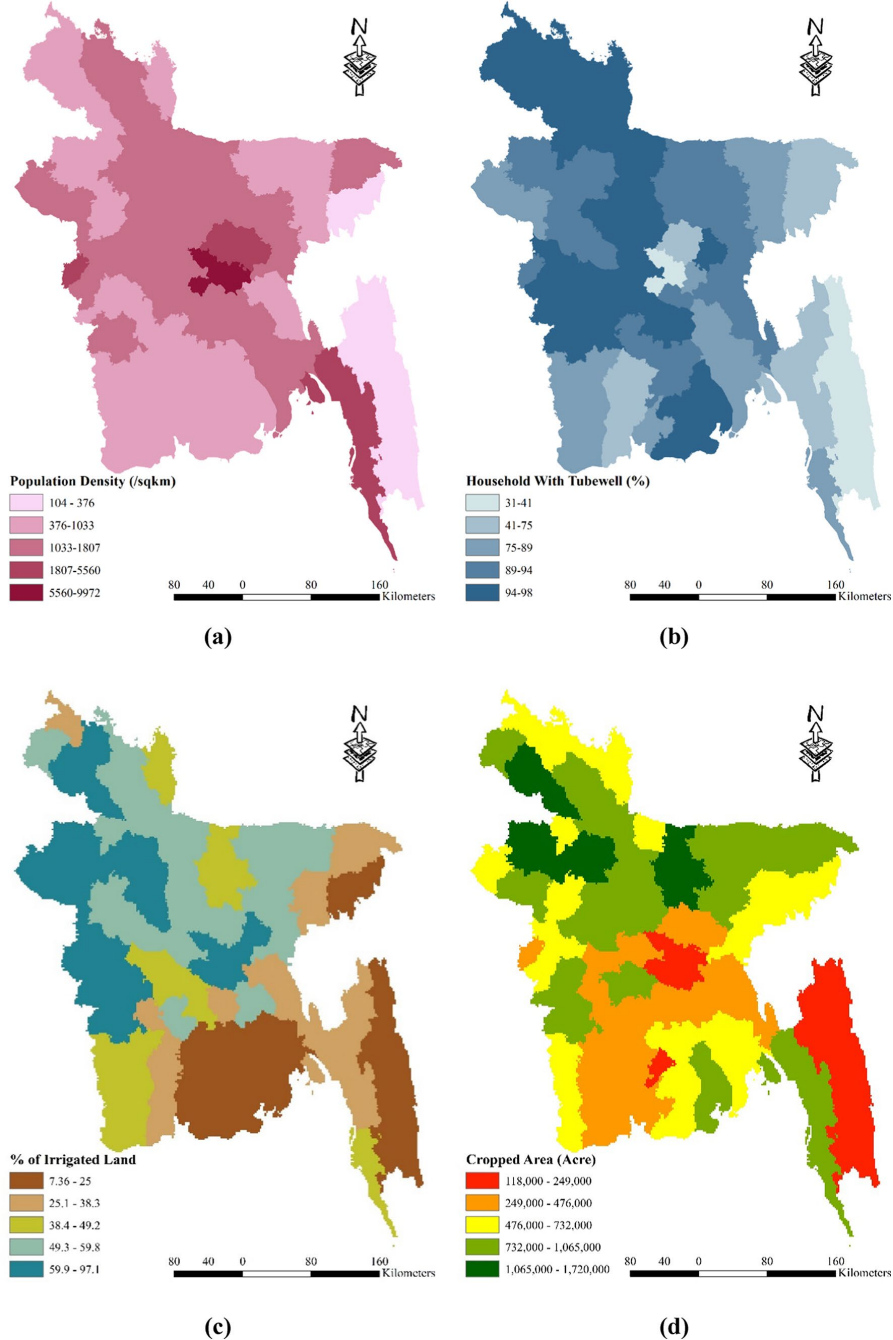

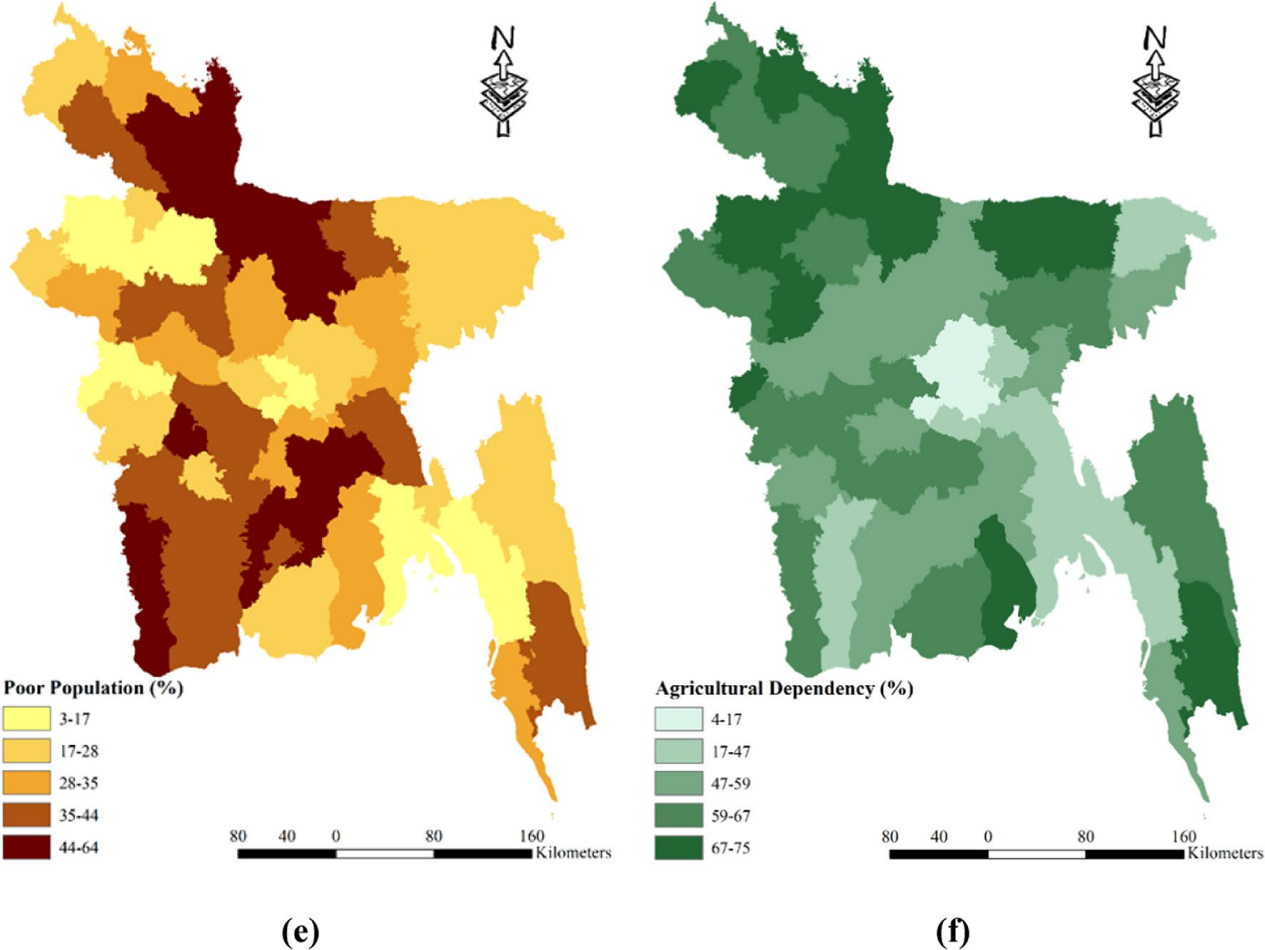
Spatial distribution socio economic drought vulnerability factors: (**a**) Population Density; (**b**) Household with Tubewell; (**c**) % of Irrigated Land; (**d**) Cropped Area (**e**) Poor Population (**f**) Agricultural Dependency (%); prepared by the authors using ArcGIS software version 10.5, (https://www.esri.com/en-us/arcgis/products).

Bangladesh, the eighth-most populous country on Earth, faces various challenges due to its high population density. With a density ranging from 104 to 9972 people per square kilometer, the demand for water resources is significantly increased. The city of Dhaka stands as the most populous area, accommodating an astonishing 9971.37 inhabitants per square kilometer. In terms of population density, there are five categorized zones, namely 104–376, 374–1033, 1033–1807, 1807–5560, and 5560–9972. Among these, the districts of Dhaka and Narayanganj face the greatest risk due to their high population density. On the other hand, Rangamati, Bandarban, Khagrachhari, and Maulvibazar are classified as low-risk areas (see Fig. 6 (a)). Furthermore, the likelihood of drought occurrences is inversely related to the percentage of households equipped with tube wells. The percentage of households with tube wells in Bangladesh ranges from 31 to 98% and is divided into five zones: 31–41%, 41–75%, 75–89%, 89–94%, and 94–98% (Table 2). The districts of Dhaka and Bandarban are particularly vulnerable, as they have fewer than 40% of households with tube wells. Conversely, 18 districts are considered less prone to drought since over 95% of households have access to tube wells (refer to Fig. 6 (b)). It is worth noting that the impact of drought hazards tends to be more severe on impoverished communities. The irrigation land of Bangladesh is classified into five classes as well Fig. 6 (c). The cropped area of Bangladesh is classified into five classes as well Fig. 6 (d). The poor population in Bangladesh is classified into five categories: 3–17%, 17–28%, 28–35%, 35–44%, and 44–64%. Six districts, namely Kurigram, Barisal, Shariatpur, Jamalpur, Chandpur, and Mymensingh, have over 50% of their population living in poverty, making them more susceptible to drought. Conversely, three districts, namely Kushtia, Noakhali, and Chittagong, have less than 15% of their population living in poverty, resulting in lower vulnerability to drought (see Fig. 6 (e)). Additionally, the likelihood of drought occurrences decreases with a higher percentage of the population engaged in agriculture. The study area in Bangladesh exhibits a range of 4 to 75% agriculture-dependent individuals, classified into five zones: 4–27%, 27–47%, 47–59%, 59–67%, and 67–75%. Four districts, namely Dhaka, Narayanganj, Gazipur, and Chittagong, have less than 20% of their population reliant on agriculture, rendering them more vulnerable to drought. Conversely, nine districts with a population consisting of over 70% agriculture-dependent individuals are considered the least vulnerable to drought (see Fig. 6 (f)). A low Agriculture Dependency % is an important element in socioeconomic drought susceptibility because of its complex relationships with economic diversification, income inequality, food security, resource allocation, and cultural dynamics within a community. When a smaller segment relies on agriculture, economic risks emerge because alternative sectors may also be prone to drought. This can result in income inequities, affecting socioeconomic equity, and local agricultural output, especially in low-dependency areas. Moreover, lesser population engaged in agriculture will also mean that the demand of water for irrigation is also lesser, which make those areas less vulnerable to drought. Also, governments may devote fewer resources to regions with low agricultural dependency, ignoring unique concerns, and movements away from traditional farming practices associated with low dependency can have an impact on social fabric and cultural identity^87^.

In summary, Bangladesh’s population density and its distribution across different districts play a crucial role in determining their vulnerability to drought hazards. The presence of tube wells, poverty levels, and reliance on agriculture further contribute to the susceptibility of specific districts to drought. Understanding these dynamics is essential for implementing targeted measures and interventions to mitigate the impact of drought and safeguard the well-being of the affected communities.

### Weight of sub-factors, factors and individual drought maps

In this modeling study, 24 indicators across four categories—socio-economic, meteorological, hydrological, and agricultural—were evaluated to assess drought vulnerability (Table 2). The weights of these indicators were derived from literature, with specific emphasis on the pairwise comparison matrices provided in the tables.

For meteorological drought, indicators such as rainfall and land surface temperature were highlighted with eigenvalues of 0.44 and 0.341, respectively, indicating their significant role in drought assessment (Table 3). Specific humidity and evapotranspiration were assigned lower eigenvalues of 0.143 and 0.076, respectively, reflecting their lesser but still important impact. The meteorological category exhibited a λmax of 4.21 and a consistency ratio (CR) of 7.7%. In the agricultural drought category, diverse indicators such as curvature, land cover, and soil type, among others, were examined. Notably, crop intensity and most used irrigation methods received higher eigenvalues of 0.237 and 0.187, respectively, underscoring their critical importance (Table 4). This category’s λmax and CR were calculated as 9.674 and 5.8%, respectively. Hydrological drought analysis revealed that drainage density had the highest eigenvalue of 0.45, demonstrating its pivotal role, while digital elevation models (DEM) had the lowest eigenvalue of 0.077 (Table 5). This category’s λmax and CR stood at 4.08 and 3.2%, respectively. Socio-economic drought factors such as population density and irrigated land area were assessed, with population density achieving the highest relative importance of 0.311, contrasting with households with tubewell, which had a lower relative importance of 0.172 (Table 6). The socio-economic category had a λmax of 6.09 and a CR of 1.5% (Table 7).

**Table 3:** Pairwise comparison matrix for meteorological drought indicators.

Factors	(1)	(2)	(3)	(4)	Eigenvalues
(1) Rainfall	1				0.44
(2) Land Surface Temperature	0.50	1			0.341
(3) Specific Humidity	0.33	0.25	1		0.143
(4) Evapotranspiration	0.25	0.25	0.33	1	0.076
λmax = 4.21	CR = 7.7%

**Table 4:** Pairwise comparison matrix for agricultural drought indicators.

Factors	(1)	(2)	(3)	(4)	(5)	(6)	(7)	(8)	(9)	Eigenvalues
(1) Curvature	1									0.028
(2) Land Cover	5	1								0.164
(3) Soil Type	6	0.33	1							0.078
(4) Geology	3	0.25	1	1						0.064
(5) Morphology	2	0.25	1	0.50	1					0.052
(6) NDVI	4	0.50	2	3	3	1				0.127
(7) Crop Intensity	4	3	2	3	3	2	1			0.237
(8) Most Used Irrigation	4	3	2	3	3	2	0.50	1		0.187
(9) Soil Texture	4	0.33	1	1	1	0.33	0.20	0.50	1	0.063
λmax = 9.674	CR = 5.8%

**Table 5:** Pairwise comparison matrix for hydrological drought indicators.

Factors	(1)	(2)	(3)	(4)	(5)	Eigenvalues
(1) Groundwater	1					0.412
(2) Drainage Density	0.50	1				0.256
(3) Flow Accumulation	0.33	0.50	1			0.152
(4) Slope	0.25	0.33	0.50	1		0.102
(5) DEM	0.25	0.33	0.50	0.50	1	0.077
λmax = 4.08	CR = 3.2%

**Table 6:** Pairwise comparison matrix for socio-economic drought indicators.

Factors	(1)	(2)	(3)	(4)	(5)	(6)	Eigenvalues
(1) Population Density	1						0.311
(2) Households with Tubewells	0.5	1					0.172
(3) Irrigated Area	0.5	1	1				0.221
(4) Crop Area	0.5	1	0.5	1			0.154
(5) Agriculture-dependent People	0.33	0.5	0.33	0.5	1		0.079
(6) Poor People	0.25	0.33	0.25	0.33	1	1	0.063
λmax = 6.09	CR = 1.5%

**Table 7:** Pairwise comparison matrix for overall drought indicators.

Factors	(1)	(2)	(3)	(4)	Eigenvalues
(1) Meteorological Drought	1				0.507
(2) Socio-economic Drought	0.25	1			0.214
(3) Hydrological Drought	0.50	0.50	1		0.178
(4) Agricultural Drought	0.25	0.50	0.50	1	0.101
λmax = 4.18	CR = 6.8%				

### Meteorological drought map

**Fig. 7:**
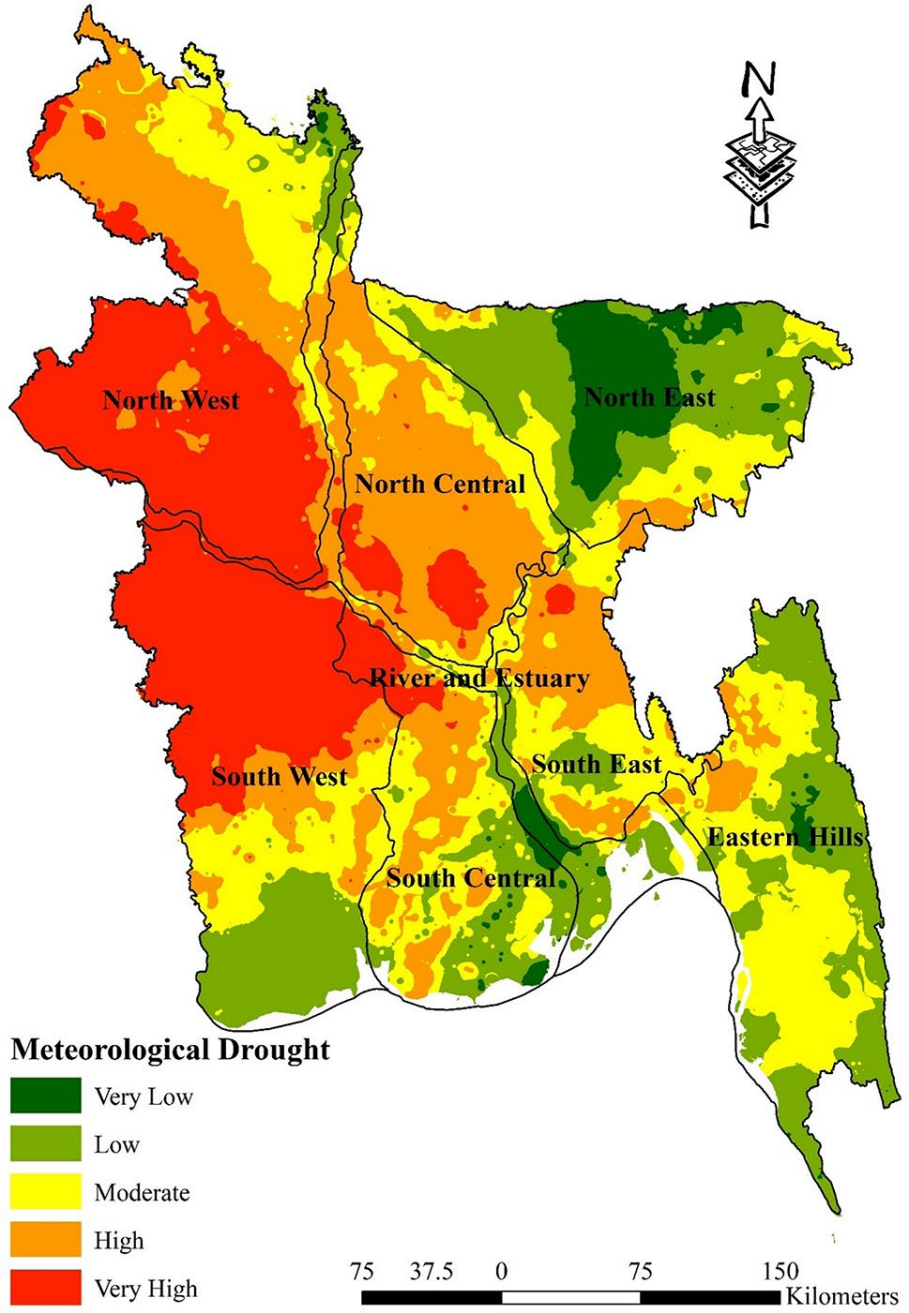
Meteorological Drought Map; prepared by the authors using ArcGIS software version 10.5, (https://www.esri.com/en-us/arcgis/products).

Table 8 shows the percentage of metrological drought vulnerable zones. There is a notable prevalence of moderate vulnerability (48.03%) in the Eastern Hills region, but no locations are categorized as having very high vulnerability (0.00%). Conversely, the North Central region experiences a significant impact from high vulnerability (66.32%), whereas low vulnerability is present to a small extent (2.80%). The region of the North East exhibits a notable proportion of low vulnerability (46.67%) and lacks any instances of very high risk. The North West region exhibits a notably high susceptibility rate of 48.74%, whereas the prevalence of very low vulnerability is almost insignificant at 0.26%. The River and Estuary region exhibits a predominantly low vulnerability rate of 32.24%, whereas instances of very high vulnerability are rather few, accounting for only 11.91%. South Central exhibits a moderate level of susceptibility, accounting for 33.55% of the total, while a lower level of very high vulnerability is observed at 9.77%. The South East region experiences a significant level of susceptibility, accounting for 43.51% of the total, while the lowest level of vulnerability is observed at 0.75%. The South West region has a notably elevated level of vulnerability, with a rate of 49.13%, while no locations are classified as having extremely low or low susceptibility, with a rate of 0.00%. This analysis highlights the diverse vulnerability of Bangladesh’s hydrological regions to meteorological drought, emphasizing particular areas of concern and potential resilience (Fig. 7).

**Table 8:** Percentage of meteorological drought vulnerable zones.

Vulnerable zones	Hydrological region							
	Eastern Hills	North Central	North East	North West	River and Estuary	South Central	South East	South West
Very Low	1.60%	0.00%	26.39%	0.26%	6.98%	2.84%	0.75%	0.00%
Low	44.03%	2.80%	46.67%	2.73%	32.24%	25.41%	12.66%	20.29%
Moderate	48.03%	18.99%	23.40%	18.58%	23.08%	33.55%	40.94%	17.21%
High	6.34%	66.32%	3.55%	29.70%	25.79%	28.42%	43.51%	13.37%
Very High	0.00%	11.88%	0.00%	48.74%	11.91%	9.77%	2.14%	49.13%

### Agricultural drought map

**Fig. 8:**
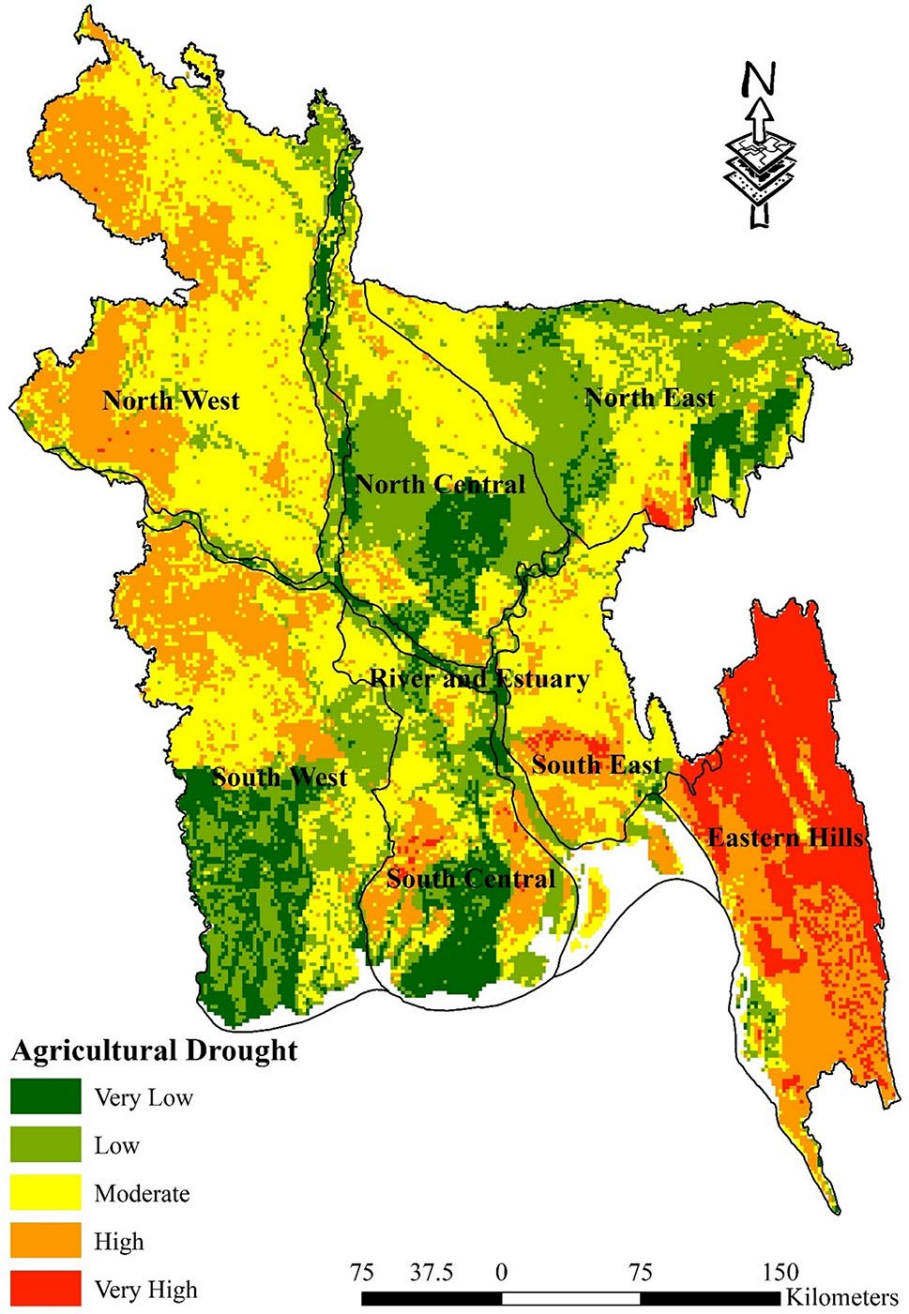
Agricultural Drought Map; prepared by the authors using ArcGIS software version 10.5, (https://www.esri.com/en-us/arcgis/products).

Table 9 displays the proportion of agricultural areas that are susceptible to drought. The Eastern Hills region experiences the highest level of risk, accounting for 54.39% of the total, while the lowest level of vulnerability, at a mere 0.44%, is observed. It is worth noting that the North West region demonstrates the highest proportion of moderate vulnerability among all regions, with a rate of 60.74%. This stands in contrast to the relatively low vulnerability rate of 0.08% observed in other regions. By contrast, the South East region distinguishes itself by having 63.02% of its land area classified as moderate vulnerability, which is the most among all categories. This is in contrast to the very low vulnerability rate of 0.69%. Although the South West does not have any places classified as highly vulnerable, a significant proportion of its territory, specifically 33.37%, falls into the intermediate category. In contrast, the North Central region, which does not have any places with a high level of vulnerability, exhibits its most notable vulnerability in the low category, accounting for 39.52%. The presence of varying levels of vulnerability highlights the urgent requirement for agricultural drought mitigation strategies that are tailored to specific regions. This is particularly crucial in areas such as the North West and South East, where moderate vulnerability is prevalent. This indicates a pressing necessity for targeted interventions aimed at enhancing resilience against the impacts of agricultural drought (Fig. 8).

**Table 9:** Percentage of agricultural drought-vulnerable zones.

Vulnerable zones	Hydrological region							
	Eastern Hills	North Central	North East	North West	River and Estuary	South Central	South East	South West
Very Low	0.44%	16.27%	9.80%	0.08%	21.85%	22.74%	0.69%	18.67%
Low	2.31%	39.52%	46.69%	6.79%	41.02%	26.84%	6.01%	25.20%
Moderate	5.08%	37.89%	38.92%	60.74%	26.35%	30.02%	63.02%	33.37%
High	37.78%	6.32%	3.60%	32.31%	10.72%	19.23%	25.84%	22.77%
Very High	54.39%	0.00%	0.99%	0.08%	0.05%	1.18%	4.44%	0.00%

### Hydrological drought map

**Fig. 9:**
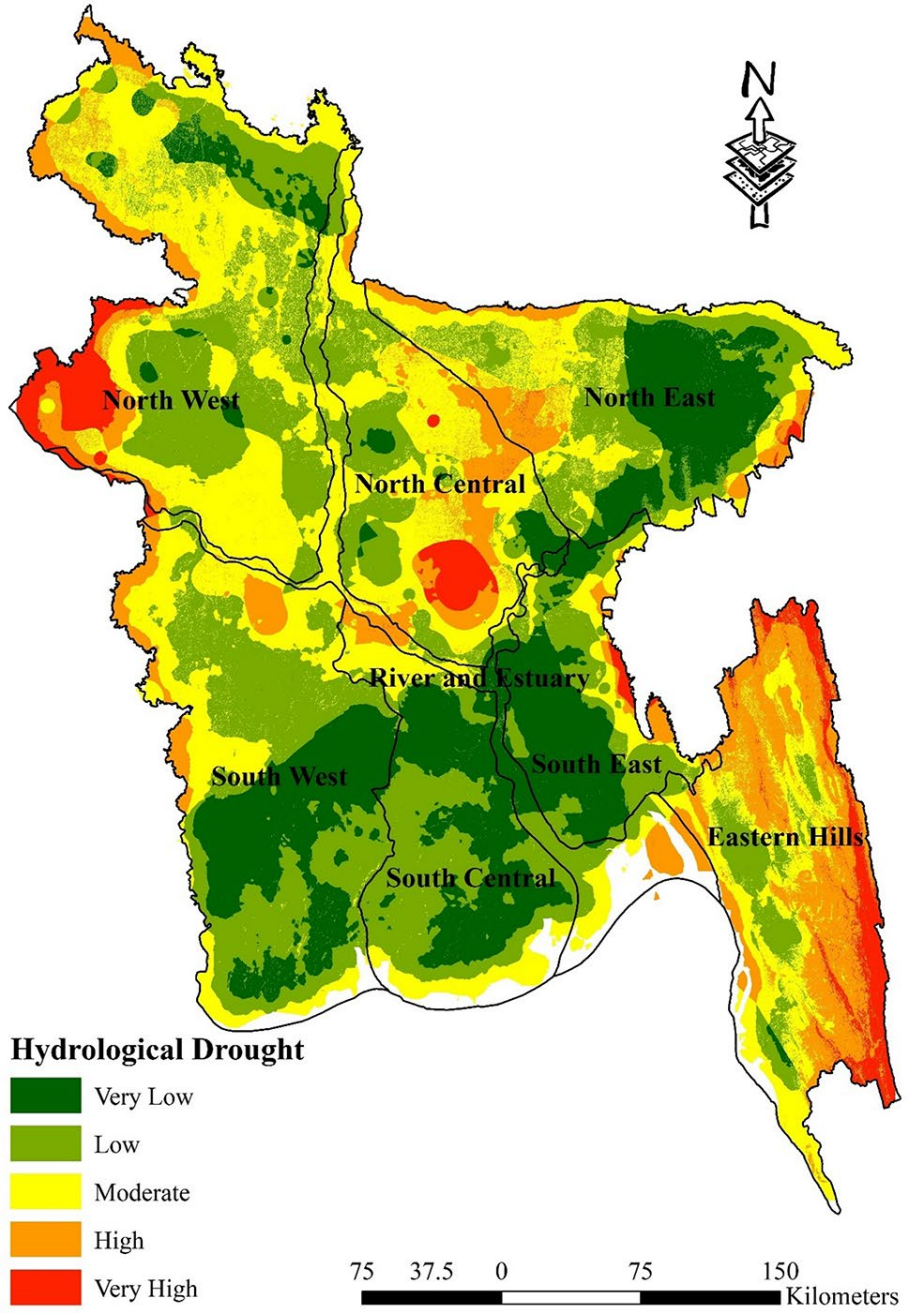
Hydrological Drought Map; prepared by the authors using ArcGIS software version 10.5, (https://www.esri.com/en-us/arcgis/products).

Table 9 displays the proportion of areas that are susceptible to hydrological drought. In the Eastern Hills, there is a significant vulnerability rate of 42.35%. However, the entrance point for very low vulnerability is very low at 0.38%, while moderate vulnerability is found at 29.58%. The North Central region has a combination of susceptibility, with moderate vulnerability accounting for 41.85% and very low vulnerability accounting for 4.74%. The distribution of drought risk in the North East and North West areas exhibits a balanced pattern, with the low and moderate categories reaching their respective peaks. This suggests the presence of a nuanced drought risk. The intermediate group has a dominant presence in the River and Estuary, accounting for 39.13% of the total, while the extremely high category has a negligible presence of 2.28%. The terrain of South Central is characterised by a notable low vulnerability rate of 44.07%, while the extremely high vulnerability rate is negligible at 0.00%. Additionally, there is a moderate vulnerability rate of 15.09%. The South East region exhibits a very low vulnerability rate of 49.02%, indicating a lower chance of drought. In contrast, the South West region largely has a low vulnerability rate of 41.06%, with extremely high vulnerability being nearly nonexistent at 0.01%. This indicates a range of risk levels, with intermediate vulnerability observed at 22.74%. The depiction highlights the intricate dynamics of hydrological drought susceptibility throughout Bangladesh, emphasising the necessity for customised measures to mitigate risks that encompass both the most severe risks and the intermediate vulnerabilities that impact a substantial percentage of the terrain. Understanding these vulnerability patterns can inform targeted mitigation and adaptation strategies to address the potential impacts of drought (Fig. 9; Table 10).

**Table 10:** Percentage of hydrological drought-vulnerable zones.

Vulnerable zones	Hydrological region							
	Eastern Hills	North Central	North East	North West	River and Estuary	South Central	South East	South West
Very Low	0.38%	4.74%	30.83%	4.92%	13.21%	39.99%	49.02%	29.46%
Low	13.97%	28.25%	28.99%	35.45%	32.22%	43.07%	31.56%	41.06%
Moderate	29.58%	41.85%	28.35%	42.70%	39.13%	15.09%	11.76%	22.74%
High	42.35%	18.34%	11.20%	10.28%	13.16%	1.85%	5.71%	6.74%
Very High	13.72%	6.83%	0.62%	6.66%	2.28%	0.00%	1.96%	0.01%

### Socio-economic drought

**Fig. 10:**
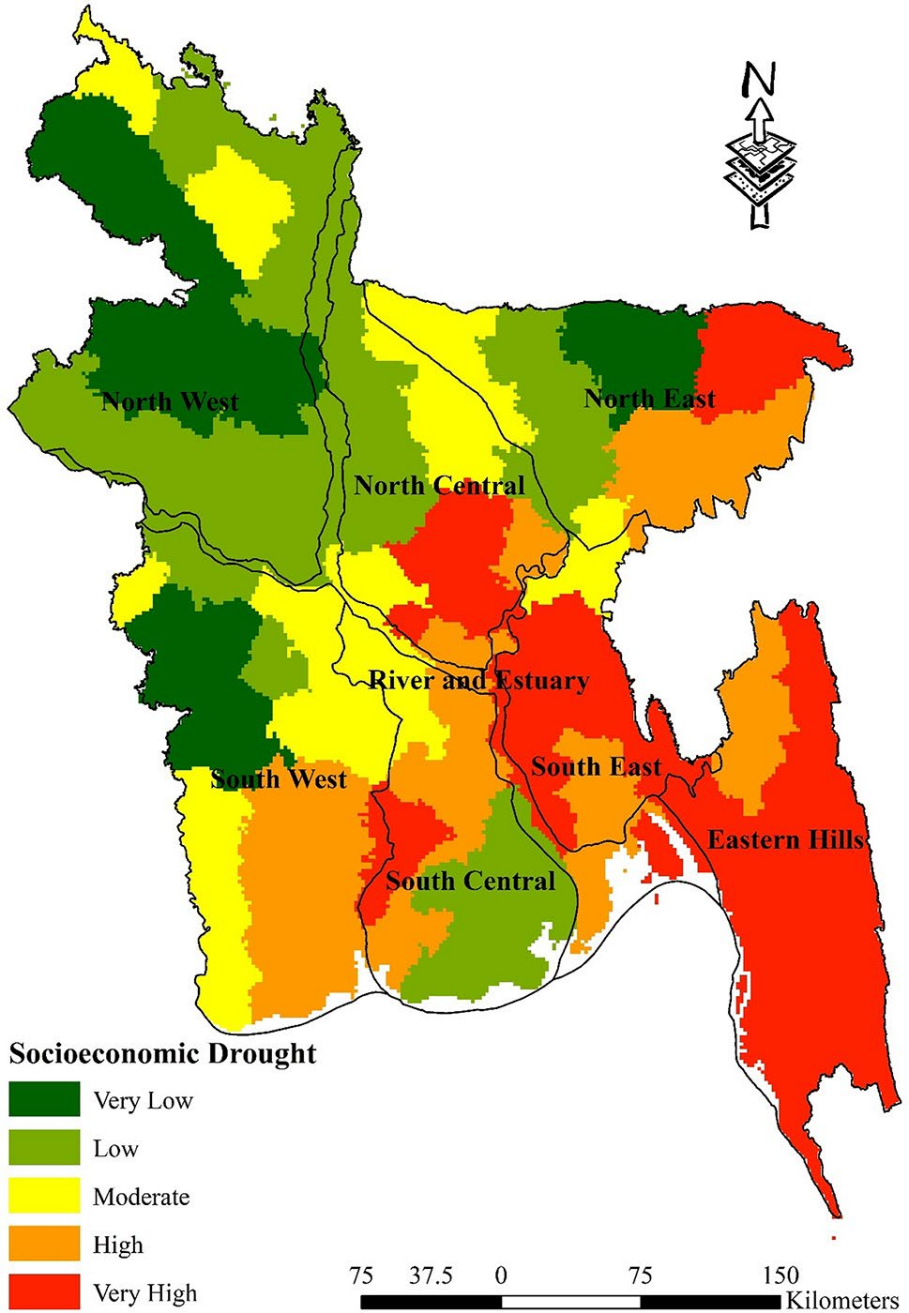
Socio-economic Drought Map; prepared by the authors using ArcGIS software version 10.5, (https://www.esri.com/en-us/arcgis/products).

Table 11 displays the proportion of socioeconomic regions that are susceptible to drought. In the Eastern Hills region, a significant proportion of 85.45% of the land area is classified as “Very High” vulnerability, hence emphasizing the pronounced hazards encountered by this particular area. In contrast, the North Central region has a notable proportion of 38.51% of its land area classified as “Low” vulnerability. This suggests that the region is comparatively less impacted by socioeconomic drought factors. However, it is important to acknowledge that the region still possesses vulnerabilities that warrant attention. The North East region, which has a significant 23.66% of its population classified as “Low” susceptibility, indicates a well-balanced and prudent strategy for managing drought risk. Conversely, the North West region does not exhibit any areas categorized as “High” susceptibility, thus focusing on its other vulnerabilities. The River and Estuary region has a notable prevalence of vulnerability, with 40.72% falling under the “Low” category. This is contrasted with a concerning 20.90% falling under the “Very High” category, indicating the intricate relationship between water availability and socioeconomic stability. The sensitivity of the South-Central region is notably high, with a percentage of 33.44%. This indicates the presence of distinct socioeconomic issues that contribute to the increased susceptibility to drought. In contrast, the South East region exhibits a notable prevalence of 65.95% categorized as “Very High” vulnerability, highlighting significant obstacles that necessitate prompt and focused actions. Furthermore, the South West region exhibits a significant 31.80% of its population falling into the “High” vulnerability group, while only a comparatively small 1.97% falls into the “Very High” category. This indicates a combination of vulnerabilities that require sophisticated strategies for mitigating drought and providing socioeconomic assistance. This comprehensive analysis of socioeconomic drought vulnerability in Bangladesh underscores the imperative for region-specific approaches that effectively tackle the distinct problems and vulnerabilities of each locality. It sheds light on places characterized by significant risk as well as those that hold promise for fostering resilience (Fig. 10).

**Table 11:** Percentage of socioeconomic drought-vulnerable zones.

Vulnerable zones	Hydrological region							
	Eastern Hills	North Central	North East	North West	River and Estuary	South Central	South East	South West
Very Low	0.00%	0.07%	18.35%	39.05%	3.66%	0.00%	0.00%	21.96%
Low	0.00%	38.51%	23.66%	49.12%	40.72%	41.26%	0.16%	9.63%
Moderate	0.00%	26.60%	15.63%	11.83%	7.99%	14.55%	12.46%	34.64%
High	14.55%	10.45%	25.41%	0.00%	26.73%	33.44%	21.43%	31.80%
Very High	85.45%	24.37%	16.94%	0.00%	20.90%	10.74%	65.95%	1.97%

#### Overall drought vulnerability map

**Fig. 11:**
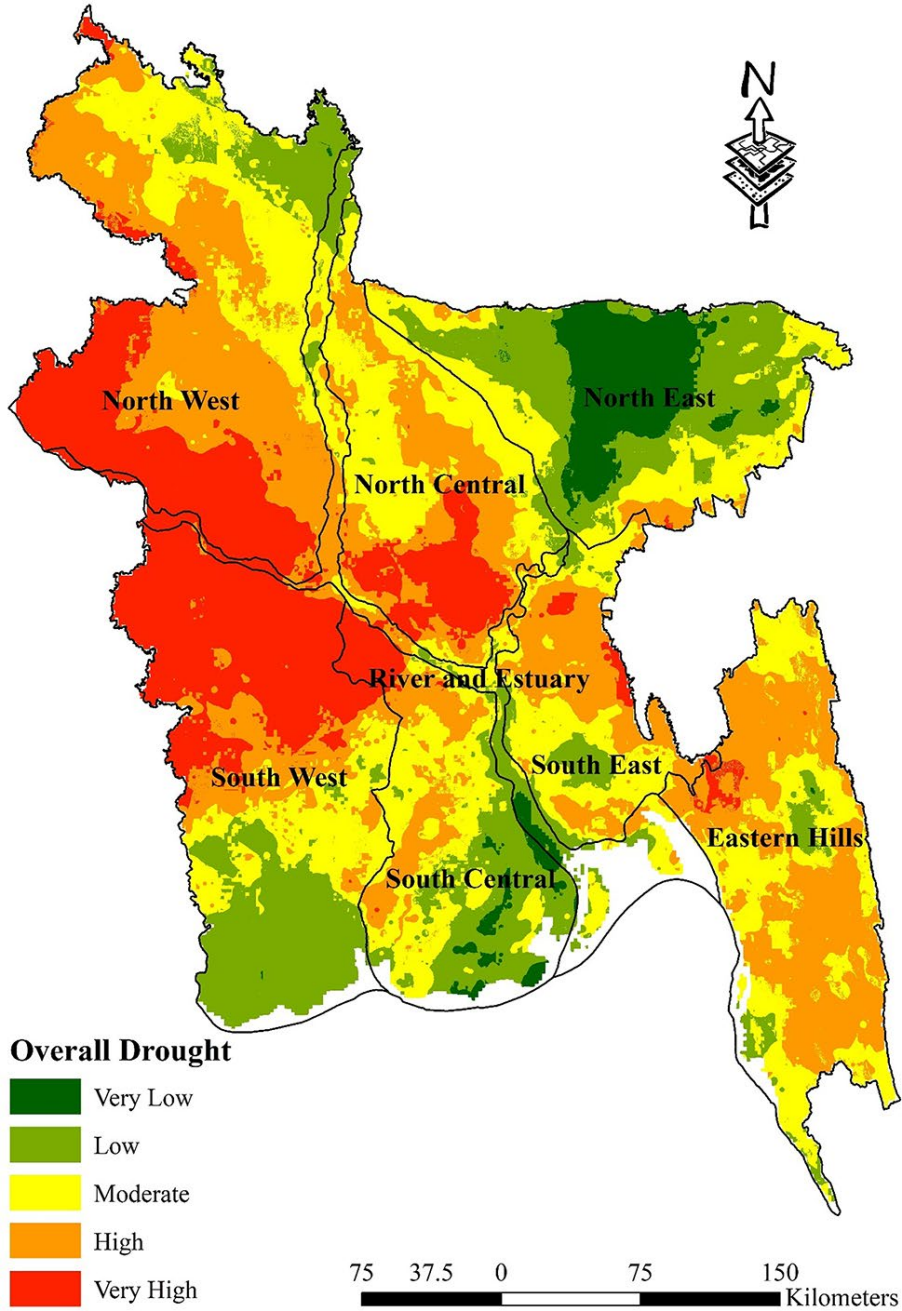
Overall Drought Vulnerability Map; prepared by the authors using ArcGIS software version 10.5, (https://www.esri.com/en-us/arcgis/products).

Table 12 displays the proportion of regions that are susceptible to drought. The Eastern Hills exhibit a significantly high vulnerability rate of 56.85%, and it has an extremely low vulnerability rate of 0.03%. The North Central region has a substantial percentage of its land area vulnerable to high levels (35.85%) and a conspicuous lack of susceptibility at very low levels. In the North East, a significant proportion (41.68%) is classified as low vulnerability, indicating a distinct risk profile in contrast to the extremely low vulnerability, which is nearly insignificant. The North West region exhibits a notable susceptibility rate of 40.39%, underscoring its significance as a crucial location for the implementation of drought management strategies. The region encompassing the River and Estuary exhibits a modest vulnerability percentage, specifically 38.44%, which suggests a well-balanced distribution across the spectrum of susceptibility. The South Central and South East regions have significant vulnerabilities, with 18.99% and 39.60% respectively, highlighting the varied effects of drought throughout the nation. The South West region exhibits a notable level of sensitivity (41.06%), indicating a pronounced susceptibility to drought hazards. This detailed analysis highlights the diverse susceptibility to drought across Bangladesh, underscoring the necessity of implementing region-specific approaches to tackle the difficulties presented by various types of droughts (Fig. 11).

**Table 12:** Percentage of overall drought-vulnerable zones.

Vulnerable zones	Hydrological region							
	Eastern Hills	North Central	North East	North West	River and Estuary	South Central	South East	South West
Very Low	0.03%	0.00%	28.57%	0.03%	4.40%	5.54%	0.00%	0.02%
Low	5.10%	2.85%	41.68%	8.56%	25.37%	31.37%	11.89%	26.01%
Moderate	36.34%	40.65%	26.10%	23.06%	38.44%	36.94%	39.60%	17.52%
High	56.85%	35.85%	3.56%	40.39%	21.19%	18.99%	44.42%	15.38%
Very High	1.68%	20.66%	0.09%	27.96%	10.60%	7.16%	4.09%	41.06%

#### Validation

**Fig. 12:**
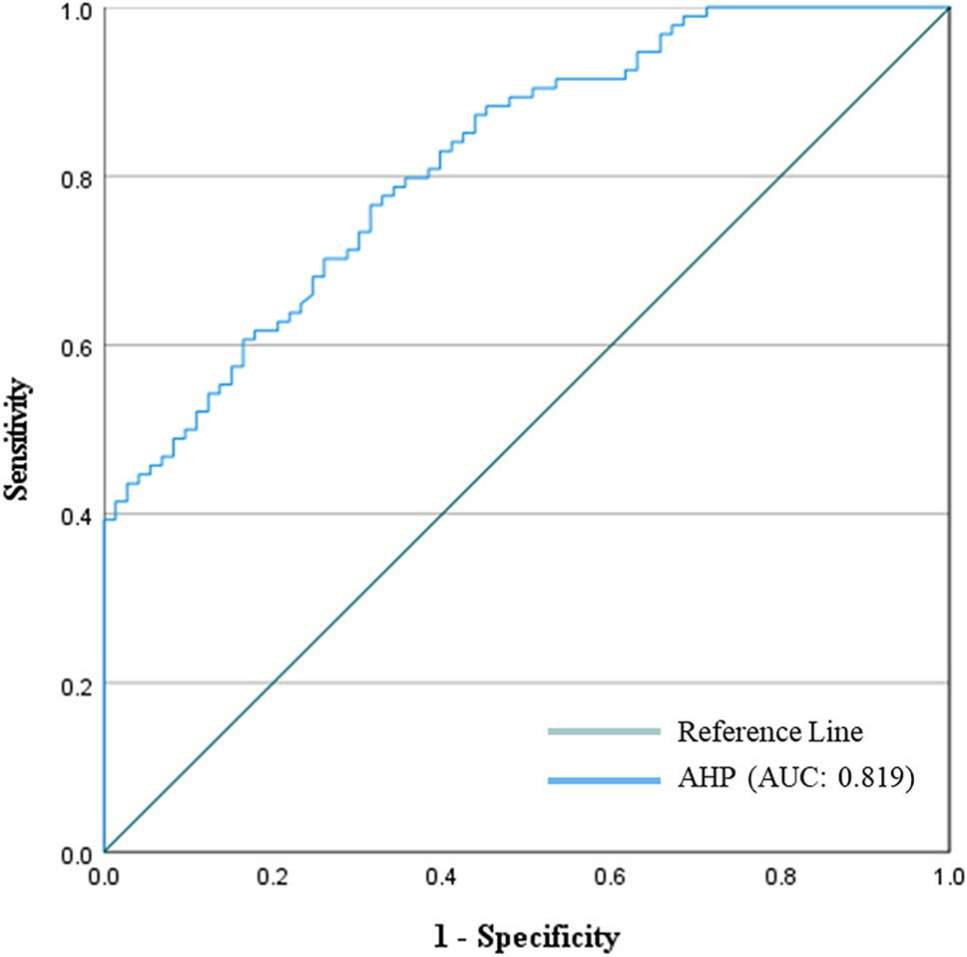
The ROC curve for overall drought vulnerability.

In order to assess the performance of predictive models, it is crucial to conduct testing or validation procedures. In this particular study, we adopted the ROC curve method for model validation, specifically focusing on the AUC. This method serves as a fundamental component of probability-based mapping and plays a vital role in the success of most statistical or probabilistic models. The ROC curve, widely recognized and preferred in the field, has been employed in numerous studies, including reference^47^. The resulting AUC value, which stands at 0.819, demonstrates an acceptable level of performance (as depicted in Fig. 12). This metric serves as a reliable indicator of the model’s predictive ability, suggesting that the model’s predictions align reasonably well with the observed data.

## Discussion

Drought is a global issue that may have disastrous consequences for people’s lives and livelihoods, including agriculture and forests. To identify the overall drought vulnerability of Bangladesh, a combination of geospatial techniques and the AHP has been used. A total of 20 criteria were chosen from among four categories of drought: meteorological, agricultural, hydrological, and socioeconomic.

First of all, pairwise matrices have been used to reflect the relative importance or preference given to one indicator compared to another. The relationship between several meteorological drought indicators is elucidated by the pairwise comparison matrices presented in Table 3. The significance of rainfall in measuring meteorological drought sensitivity is shown by its emergence as the most crucial element. In descending order, the significance of land surface temperature, specific humidity, and evapotranspiration is quantified. The meteorological drought map indicates that the susceptibility to meteorological drought in the eastern hill’s region, which includes districts such as Sylhet and Moulvibazar, ranges from extremely low to moderate. This indicates a relatively stable situation in this regard. The north west region, encompassing districts like Panchagarh and Rajshahi, is most susceptible to drought-related risks. In a similar vein, the north central region and River and Estuary, which includes districts like Dhaka, Faridpur, Gopalgonj, Bogura, and Natore, exhibit diverse degrees of susceptibility to meteorological drought vulnerability; thus, customised interventions are imperative to mitigate these risks. It is noteworthy that the south west region, encompassing districts such as Khulna and Satkhira, exhibits the least susceptibility to meteorological drought-related difficulties, indicating a relatively robust topography (Fig. 7). Table 4, sheds light on the relative importance of various factors contributing to agricultural drought vulnerability in Bangladesh. Curvature emerges as a significant indicator, suggesting its pivotal role in assessing agricultural drought susceptibility. This is followed by land cover, soil type, and geology, each contributing to the understanding of agricultural drought risks in different regions. Notably, the comparison underscores the importance of factors such as NDVI, crop intensity, and irrigation methods, providing insights into the diverse aspects influencing agricultural drought vulnerability across various districts. Table 9 sheds light on how Bangladesh’s several hydrological zones distribute the risk of agricultural drought. It shows that there are large differences in different regions’ degrees of vulnerability, with some having high levels of vulnerability and others having relatively lower levels of susceptibility to agricultural drought. For example, the Eastern Hills region’s sensitive zones have the largest percentage of very high vulnerability, which denotes the highest danger of agricultural drought. Comparably, the North West area exhibits a great deal of risk as well, especially when it comes to zones of moderate to high susceptibility. On the other hand, locations with lower degrees of vulnerability, like the South West, appear to have a more robust agricultural environment (Fig. 8). On the contrary river and estuary region comprise of high to low vulnerable zones. Previous studies^69^ showed that Dhaka, Manikganj, Faridpur, Gazipur, Sirajganj, Tangail, Rajbari, Faridpur, Pirojpur, Patuakhali, and portions of Gaibandha, Sherpur, Chapainawabganj, Rangpur, Dinajpur, Khushtia, Narail, Cumilla, Kishorganj, and Bhola are among the districts that are particularly vulnerable to agricultural drought^69^. Both the amount of rainfall and the soil’s ability to retain water were poor in such regions, which is quite similar to our study. Table 5 shows that subfactors like groundwater and drainage density have higher relative importance compared to other subfactors in the hydrological drought section. Regarding hydrological drought, the majority of the hilly regions fall into the very vulnerability group. The hydrological drought vulnerability across Bangladesh is presented in Table 10. Evidence indicates that the risk levels in the Eastern Hills region vary, with a significant number of zones exhibiting moderate to high sensitivity. Furthermore, the North Central and South East regions display different levels of susceptibility, with a significant proportion of zones classified as moderately susceptible. Conversely, places such as the South West exhibit comparatively lower levels of vulnerability, suggesting a more robust hydrological environment in these areas. Considering the hydrological settings, the geographic distribution of groundwater and the varying elevation levels contribute to a significant hydrological drought in the central region, namely in Dhaka and the Chittagong Hill Tracts (Fig. 9). Notably, the North West region shows varying vulnerability, with certain zones demonstrating moderate vulnerability^88^. Table 6 comparison matrix highlights factors such as population density, households with tubewells, irrigated area, crop area, agriculture-dependent people, and poor people. Each factor plays a crucial role in understanding socio-economic drought risks across different regions. In the case of the socio-economic drought map, there are notable differences in the vulnerability of various locations to socioeconomic drought, with some areas displaying a high level of vulnerability and others a relatively lower level. For example, a significant portion of the land is covered by susceptible zones in the North East and North West, which suggests increased risks of socioeconomic drought. On the other hand, locations with lower degrees of vulnerability, such South Central and the Eastern Hills, may have a more resilient socioeconomic environment (Fig. 10). Finally, Table 7 shows that factors like meteorological drought and socioeconomic drought have higher relative importance compared to other droughts. In all the cases, the value is less than 10%, so it can be said that the overall comparison matrix is acceptable for all the criteria and factors. reveals significant variations in vulnerability levels among different regions, with certain areas exhibiting high vulnerability while others demonstrate lower susceptibility to drought. For instance, the overall drought vulnerability map shows that vulnerable zones in the North Central region and wester zone (north west and south west), encompass a substantial percentage of the total area, indicating elevated risks of overall drought. Similarly, districts in the South-Central region, such as Dhaka and Narayanganj, also exhibit notable vulnerability to overall drought. Conversely, regions such as the Eastern Hills, which include districts like Sylhet and Moulvibazar, and the South West, comprising districts like Khulna and Satkhira, display relatively lower vulnerability levels, suggesting a more resilient landscape in these areas (Fig. 11). River and estuary geomorphology is more complicated and dynamic, and natural disasters including storm surges, tropical cyclones, sea level rise, erosion, and accretion have changed it over the past few hundred years^89–91^. Thus, river and estuary drought vulnerability is mostly moderate (38.44%). Study done by^92,93^ have claimed that the western region of the country faces a very high level of drought. On the other hand, previous research showed the north-western part of the country faces a very high level of drought. The higher occurrence of droughts in the northwestern part of the country is due to the high annual variability of rainfall in the region^94^. Gazipur, Narayanganj, and Dhaka are mainly industrial hubs of the country due to their huge populations, and the use of groundwater levels are also very high in these regions. Moderate drought vulnerability has been found in some portions of Thakurgaon, Gaibandha, Jamalpur, Khagrachori, Bandarban, etc.^95^. Low and very low drought vulnerability areas are observed in Sylhet Division and the southern portion of this country^92,93,95^. It is because the rainfall and geomorphic location play an important role in reducing drought in these regions^96^. So it can be said that most of the studies done in these region are quite similar to our study. This study’s findings were also efficiently verified using ROC and AUC techniques. The most widely used and accepted methods for verification are the ROC and AUC procedures. Most research employed this method to check the results of the drought^97,98^. The overall accuracy was over 80% which is accepted all over.

There exist certain constraints to this investigation. The majority of datasets utilized in this work were updated to the greatest extent feasible. Updated datasets include agricultural dependence, soil texture, and morphology. Socioeconomic drought vulnerability has been assessed using data from the 2011 census. The elevation and slope maps were generated using a 10-m spatial resolution Digital Elevation Model (DEM). An enhanced Digital Elevation Model (DEM) with higher resolution may yield a superior result. Validation of the study’s results was conducted by employing testing datasets sourced from existing research instead of utilizing actual field data. The limitations of the study include the dependence on the availability and accuracy of data, potential biases in expert predictions for AHP weight estimation, and the static nature of the model, which may not account for changing climate and socioeconomic conditions over time.

## Conclusion

Drought poses a significant threat to Bangladesh, impacting lives, livelihoods, agriculture, and ecosystems. To comprehensively assess the country’s overall drought vulnerability, this study employed a combination of geospatial techniques and the Analytical Hierarchy Process (AHP). Twenty-four criteria from meteorological, agricultural, hydrological, and socioeconomic categories were meticulously selected for analysis.

Pairwise comparison matrices were utilized to determine the relative importance of various drought indicators. For meteorological drought, rainfall emerged as the most crucial factor, with districts in the Eastern Hills region, including Sylhet and Moulvibazar, exhibiting low to moderate vulnerability, while the North West region, including Panchagarh and Rajshahi, faced higher susceptibility. In agricultural drought, factors such as curvature and land cover played significant roles, with districts like Chapainawabganj and Rajshahi demonstrating elevated vulnerability in the North West and North Central regions. Hydrological drought vulnerability varied across regions, with subfactors like groundwater and drainage density influencing susceptibility. The Eastern Hills region showed moderate to high vulnerability, while the South West exhibited a more resilient landscape. Socioeconomic drought vulnerability was influenced by factors like population density and irrigation methods, with districts in the North East and North West regions facing heightened risks. Overall, certain districts, notably Chapainawabganj, Rajshahi, Pabna, and Faridpur in the North West and North Central regions, were identified as highly vulnerable to drought. Conversely, regions like the Eastern Hills and South West showed relatively lower vulnerability levels.

Based on the findings of the present study, the following recommendation is made: Water conservation is an important technique to minimize drought. This entails encouraging the adoption of water-saving methods at the individual, household, and industrial levels. This might involve raising awareness about the significance of lowering water usage in daily activities, advocating the adoption of effective irrigation techniques, and installing water-saving equipment. Rainwater collection is another useful approach. Communities may gather and store rainwater for future use by constructing and promoting rainwater harvesting systems. This can include building reservoirs, placing rain barrels, or promoting the use of permeable surfaces so that rainfall can replenish groundwater sources. Furthermore, effective agricultural methods are critical to dealing with drought. To reduce water usage, farmers should be encouraged to use precision irrigation systems such as drip irrigation and micro-sprinklers. Community participation plays an important role in drought mitigation measures. Individuals may be informed about the need for water conservation through public awareness programmers. To mitigate drought vulnerability in Bangladesh, preventive strategies include improving water management through rainwater harvesting and groundwater recharge, promoting drought-resistant crop varieties and sustainable agricultural practices, enhancing early warning systems, and implementing community-based adaptation measures and reforestation projects to enhance resilience against drought impacts. It is imperative to establish and execute drought management plans in cooperation with governments, non-profit organizations, and community groups, prioritizing the development of water delivery, monitoring, and emergency response systems. The outcomes of this study will offer significant insights for government officials in making well-informed judgements about the management and planning of national drought. Armed with a comprehensive vulnerability map, policymakers and administrators can develop and implement effective strategies to mitigate the impacts of drought and minimize associated losses. Future drought vulnerability research should focus on understanding climate change’s effects, analyzing socioeconomic aspects, and establishing effective risk management measures. Participatory research and stakeholder involvement are critical for understanding local viewpoints and needs. Long-term monitoring systems are required to track changes over time and assess the efficacy of mitigation strategies. Integrating drought vulnerability with other environmental factors like land use change and ecosystem health yields a more complete picture. Furthermore, conducting economic analyses to quantify costs and investigate options such as insurance can aid in the development of long-term drought mitigation initiatives.

## Data Availability

The datasets used in the study are available from the corresponding author upon reasonable request.
